# Oxidized Low-Density Lipoprotein as a Potential Target for Enhancing Immune Checkpoint Inhibitor Therapy in Microsatellite-Stable Colorectal Cancer

**DOI:** 10.3390/antiox14060726

**Published:** 2025-06-13

**Authors:** Xiaochun Zhang, Xiaorui Ye, Heiying Jin

**Affiliations:** 1 The Second Clinical Medical College of Nanjing University of Chinese Medicine, Nanjing 210017, China; 202430161@njucm.edu.cn; 2 The Second Affiliated Hospital of Nanjing University of Chinese Medicine, Nanjing 210017, China; efy107@njucm.edu.cn

**Keywords:** oxidized low-density lipoprotein, microsatellite-stable colorectal cancer, immune checkpoint inhibitors, tumor microenvironment, metabolic reprogramming, immunosuppression, oxidative stress

## Abstract

Oxidized low-density lipoprotein (oxLDL) exhibits differential expression in microsatellite-stable (MSS) and microsatellite instability-high (MSI) colorectal cancer (CRC), highlighting its potential therapeutic role in immune checkpoint inhibitor (ICI) resistance in MSS CRC. Elevated oxLDL levels in MSS CRC contribute to tumor progression and diminish ICI efficacy by modulating metabolic reprogramming and immunosuppressive mechanisms within the tumor microenvironment (TME) by activating receptors such as LOX-1 and CD36. oxLDL triggers signaling pathways, including NF-κB, PI3K/Akt, and MAPK, leading to the expansion of immunosuppressive cells like regulatory T cells (Tregs), myeloid-derived suppressor cells (MDSCs), and M2 macrophages, while concurrently suppressing effector T cell functions. Additionally, oxLDL enhances oxidative stress and promotes fatty acid oxidation (FAO) and glycolytic metabolism, resulting in nutrient competition within the TME and establishing an immunosuppressive milieu, ultimately culminating in ICI resistance. This review systematically examines the disparities in oxLDL expression between MSS and MSI CRC and elucidates the molecular mechanisms through which oxLDL mediates ICI resistance. Furthermore, it explores potential therapeutic strategies targeting oxLDL, offering novel avenues to overcome immunotherapy resistance in MSS CRC.

## 1. Oxidative Stress in Colorectal Cancer

### 1.1. Mechanisms of Oxidative Stress Generation

Oxidative stress (OS) refers to an imbalance between reactive oxygen species (ROS) production and the antioxidant defense system, resulting in excessive ROS accumulation that damages cellular structures and functions. The precise regulation of ROS generation and elimination fundamentally governs the intricate relationship between ROS levels and cancer. In tumor cells, ROS production is elevated due to increased metabolic activity, genetic mutations, and relative hypoxia. In colorectal cancer (CRC), OS is particularly pronounced, contributing to tumor initiation, progression, and drug resistance. However, excessive ROS can also exert cytotoxic effects, triggering programmed cell death (PCD) or counteracting resistance to anticancer therapies [1,2]. The pronounced elevation of OS in CRC arises from a multidimensional interplay between the colon’s unique anatomical microenvironment and tumor-specific metabolic adaptations. Originating from intestinal epithelial cells, CRC cells exhibit rapid proliferation and heightened metabolic activity, which drive elevated oxidative DNA damage [3]. As the primary interface between the external environment and host immunity, the colonic epithelium is chronically exposed to diet-derived pro-oxidants (e.g., lipid peroxides, polycyclic aromatic hydrocarbons) and microbiota-generated metabolites (e.g., hydrogen sulfide, secondary bile acids), collectively fueling ROS generation [4].

ROS primarily originate from cellular metabolisms and external stimuli. Endogenous ROS primarily originate from mitochondrial oxidative phosphorylation (OXPHOS), where molecular oxygen undergoes incomplete reduction within the electron transport chain (ETC), generating superoxide anions (O_2_^−^) [5]. Superoxide can be produced at multiple mitochondrial sites, including Complex I (sites IQ and IF), Complex III (site IIIQo), glycerol-3-phosphate dehydrogenase, Q-cycle oxidoreductase, pyruvate dehydrogenase, and 2-oxoglutarate dehydrogenase [5]. In the mitochondrial matrix, manganese superoxide dismutase catalyzes the conversion of O_2_^−^ into hydrogen peroxide (H_2_O_2_). Similarly, in the intermembrane space or cytoplasm, copper/zinc superoxide dismutase (Cu/Zn-SOD) performs the same function [6,7]. H_2_O_2_ can further participate in Fenton reactions or react with metal ions, generating highly reactive hydroxyl radicals (•OH), thereby exacerbating OS-induced damage [8]. Additionally, under normal physiological conditions, cytochrome P450 primarily facilitates substrate oxidation, leading to water formation. However, under specific conditions—such as substrate deficiency or reduced catalytic efficiency—its enzymatic cycle may become partially uncoupled, resulting in the leakage of ROS intermediates, including O_2_^−^ and H_2_O_2_. Furthermore, cytochrome P450 can generate ROS while metabolizing various organic substrates, including lipids, steroid hormones, and xenobiotics [9,10].

**Figure 1:**
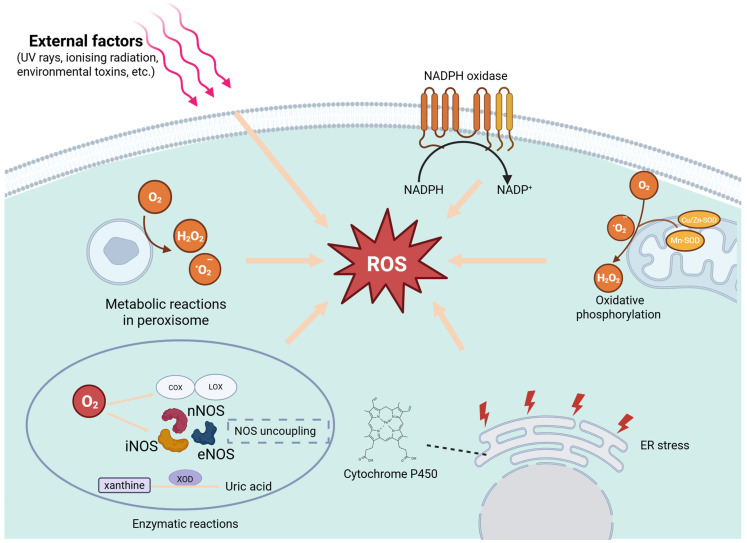
Mechanisms of ROS generation: endogenous and exogenous sources. Endogenous sources include mitochondrial electron transport chain activity, NADPH oxidase (NOX) enzymes, peroxisomal metabolism, cytochrome P450 enzyme activity, xanthine oxidoreductase (XOR), nitric oxide synthase (NOS, including nNOS, iNOS, and eNOS), and cyclooxygenase/lipoxygenase (COX/LOX). Exogenous sources encompass ultraviolet (UV) radiation, ionizing radiation, environmental toxins, and certain chemotherapeutic agents. Created in BioRender.com.

Enzymatic reactions represent a primary endogenous source of ROS. Beyond being mere byproducts of mitochondrial metabolism, ROS production is also actively driven by various enzymatic processes, particularly in specific cell types (e.g., immune cells) and under certain physiological or pathological conditions. NADPH oxidase (NOX) is an integral membrane enzyme complex primarily responsible for ROS generation. It is predominantly expressed in immune cells, such as neutrophils and macrophages, and non-immune cells, including endothelial cells, smooth muscle cells, and fibroblasts [11]. NOX facilitates the transfer of electrons from intracellular NADPH to molecular oxygen, producing O_2_^−^, which can subsequently be converted into hydroxyl radicals (•OH). Xanthine oxidoreductase, a key enzyme in purine metabolism, catalyzes the oxidation of hypoxanthine and xanthine to uric acid. During this process, molecular oxygen is reduced to O_2_^−^, further contributing to ROS accumulation [12]. Nitric oxide synthase (NOS) primarily catalyzes the conversion of L-arginine into NO. However, NOS uncoupling occurs under NOS dysfunction or deficiency conditions in substrates/cofactors (e.g., L-arginine, tetrahydrobiopterin), leading to reduced NO production and excessive ROS generation [13]. Cyclooxygenases (COXs) and lipoxygenases (LOXs), which metabolize arachidonic acid to produce inflammatory mediators such as leukotrienes and prostaglandins, also generate peroxides and O_2_^−^ as byproducts [14]. The overactivation of COXs/LOXs has been closely linked to increased ROS levels within the tumor microenvironment (TME). Additionally, peroxisomal oxidases contribute to ROS production during fatty acid β-oxidation. If the H_2_O_2_ generated in this process is not promptly degraded by catalase (CAT), OS may ensue [15]. Exogenous stimuli, such as ultraviolet radiation [16], ionizing radiation [17], and environmental toxins—including heavy metals [18], pesticides, tobacco smoke, and industrial pollutants—can directly generate ROS or induce endogenous ROS production [19]. The mechanisms of ROS generation are illustrated in Figure 1.

### 1.2. OS in the Development and Progression of CRC

**Figure 2:**
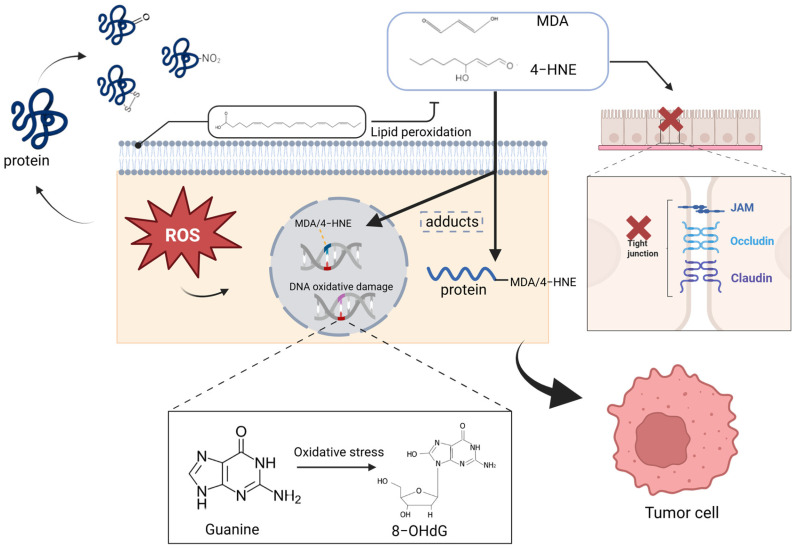
OS causes oxidative damage in DNA, lipids, and proteins, promoting the initiation and metastasis of CRC. ROS can cause DNA base oxidation and strand breaks, leading to genomic instability and mutations that promote tumorigenesis. They also react with polyunsaturated fatty acids in cell membranes, producing lipid peroxides like malondialdehyde (MDA) and 4-hydroxynonenal (4-HNE). This process disrupts membrane fluidity and integrity, increases permeability, and indirectly impairs tight junctions, further promoting ROS generation. Additionally, ROS oxidize amino acid residues in proteins, resulting in carbonylation and nitration, impairing functions of DNA repair enzymes like OGG1 and tumor suppressors such as p53, thereby accelerating cancer progression. Created in BioRender.com.

OS is considered a key driver of CRC initiation and progression. When ROS accumulate to a level where the antioxidant defense system no longer has the capacity to clear them, they can damage biomolecules such as DNA, lipids, and proteins, leading to pathological processes like abnormal cell proliferation, genomic instability, inflammation, and immune evasion, as depicted in Figure 2.

#### 1.2.1. DNA Damage: Mutations and Breaks

ROS can directly interact with DNA bases, causing oxidative damage and forming products such as 8-hydroxy-2′-deoxyguanosine (8-OHdG), which can trigger point mutations or base pair mismatches in the DNA [20]. Additionally, ROS may induce DNA double-strand breaks and single-strand breaks, leading to errors during repair processes like non-homologous end joining or homologous recombination, which can ultimately result in genomic instability [21]. Under normal circumstances, cells possess mechanisms to repair oxidative DNA damage, such as the base excision repair pathway, where repair enzymes like OGG1 (8-oxo guanine glycosylase) remove damaged bases [22,23]. If DNA damage is not repaired in time, it can lead to the accumulation of genetic mutations. Studies have shown that in five microsatellite-stable (MSS) CRC cell lines, the level of 8-OHdG was significantly elevated (approximately 33%), accompanied by defects in the repair mechanisms associated with 8-OHdG, suggesting a higher incidence of oxidative DNA damage and dysfunction in DNA repair systems in these cell lines [24]. Furthermore, ROS may activate proto-oncogenes (e.g., KRAS and MYC) or suppress tumor suppressor genes (e.g., TP53 and APC), further promoting cancer progression [25,26]. ROS can also alter DNA methylation patterns and histone modifications, thus regulating the expression of key tumor-associated genes [27].

#### 1.2.2. Lipid Damage: Lipid Peroxidation

When ROS react with polyunsaturated fatty acids in cell membranes, they abstract hydrogen atoms, generating lipid radicals (L•) and lipid peroxyl radicals (LOO•). Subsequently, LOO• reacts with oxygen molecules to form highly reactive secondary products. Among these, malondialdehyde (MDA) and 4-hydroxy-2-nonenal (4-HNE) are two key end products of lipid oxidation, commonly used to assess lipid peroxidation levels [28]. Lipid peroxidation not only disrupts the fluidity and integrity of the phospholipid bilayer, increasing membrane permeability, which leads to abnormal ion and molecular transmembrane flux and interferes with normal cellular functions, but it also triggers ROS-induced cardiolipin peroxidation [29]. This alters the mitochondrial membrane structure, weakening the stability of the ETC. Mitochondrial lipid peroxidation destabilizes Complexes I and III, increasing electron leakage and further ROS production, creating a vicious positive feedback loop [30]. Furthermore, excess ROS and their lipid peroxidation products not only inhibit the activities of antioxidant enzymes, such as GPx and SOD, thus reducing the ability to clear ROS, but they can also activate the nuclear factor kappa B (NF-κB) inflammatory signaling pathway, inducing the release of pro-inflammatory cytokines (e.g., IL-6, TNF-α, and IL-1β) [31,32,33,34]. This, in turn, continuously promotes the initiation and progression of CRC.

#### 1.2.3. Protein Damage: Oxidative Modifications

MDA and 4-HNE can covalently bind to DNA and proteins, forming adducts that induce genetic mutations and epigenetic changes [35]. Furthermore, lipid oxidation products damage the intestinal epithelial cell membranes and tight junction proteins, weakening the intestinal barrier function [36]. This makes it easier for toxic molecules to penetrate the body, triggering chronic inflammation and increasing the risk of carcinogenesis [36]. At the same time, ROS can oxidize amino acid residues in proteins, such as cysteine and methionine, altering proteins’ tertiary structure and biological activity. This leads to protein carbonylation, nitration, and abnormal disulfide bond formation, affecting their function, structure, and stability [37,38]. Studies have shown that protein nitration is closely associated with immune evasion and treatment resistance in cancer [36]. Moreover, oxidative modifications can affect the function of key regulatory proteins in signaling pathways, such as APC and β-catenin, impairing tumor suppression mechanisms or abnormally activating oncogenes [39,40]. For example, the ROS-induced oxidation of DNA repair enzyme OGG1 and tumor suppressor protein p53 significantly weakens their activity, accelerating cancer progression [41]. Additionally, the partial inhibition of antioxidant enzymes like GPx and SOD leads to further ROS accumulation, exacerbating oxidative damage [42,43].

#### 1.2.4. Key Effector Molecules of OS Drive the Development of CRC

OS and its critical pathological effector molecule, oxidized low-density lipoprotein (oxLDL), serve as pivotal drivers of colorectal carcinogenesis and progression. Lipoproteins, including low-density lipoprotein (LDL), high-density lipoprotein (HDL), and triglycerides, act as primary carriers of lipids in circulation, playing essential roles in lipid transport and metabolism [44]. In the tumor microenvironment (TME) of CRC, dysregulated lipid metabolism induces lipotoxicity, triggering oxidative stress that promotes LDL oxidation and elevates ROS levels [45]. Progressive oxidative stress perpetuates intracellular LDL oxidation, leading to the accumulation of oxLDL—a core mediator of oxidative damage. Elevated plasma oxLDL levels, observed in breast, gastric, and colorectal cancers [46,47,48], correlate with its role as a key effector of oxidative stress in CRC pathogenesis. Mechanistically, oxLDL arises from ROS-mediated (e.g., HOCl, O_2_^−^) or enzymatic (e.g., myeloperoxidase [MPO], lipoxygenase [LOX]) modification of LDL’s apolipoprotein B-100 and polyunsaturated fatty acids (PUFAs) within phospholipid layers. This oxidative modification drives oncogenic processes, including proliferation, invasion, angiogenesis, epithelial–mesenchymal transition (EMT), migration, and dysregulated autophagy [49]. Notably, the binding of oxLDL to LOX-1 receptors exhibits a positive feedback loop with ROS generation, exacerbating oxidative DNA damage [50]. These findings collectively position oxLDL not only as a biomarker of oxidative stress but also as a therapeutic target to disrupt the interplay between lipid dysregulation, oxidative damage, and tumor progression in CRC.

## 2. Expression and Regulation of oxLDL in CRC

In physiological conditions, lipoproteins maintain lipid homeostasis, supporting cell membrane construction and energy storage. However, LDL is prone to oxidative modification under OS, forming oxLDL, whose structural and functional alterations have significant pathological implications [51]. High-fat diets, smoking, and hyperglycemia can increase free radical concentrations, promoting LDL oxidation [52]. oxLDL is recognized and internalized by tumor cells and immune cells through its specific receptor, lectin-like oxidized LDL receptor-1 (LOX-1), activating downstream signaling pathways that enhance tumor cell proliferation, migration, and invasion [53].

### 2.1. Expression Characteristics of oxLDL in CRC

**Figure 3:**
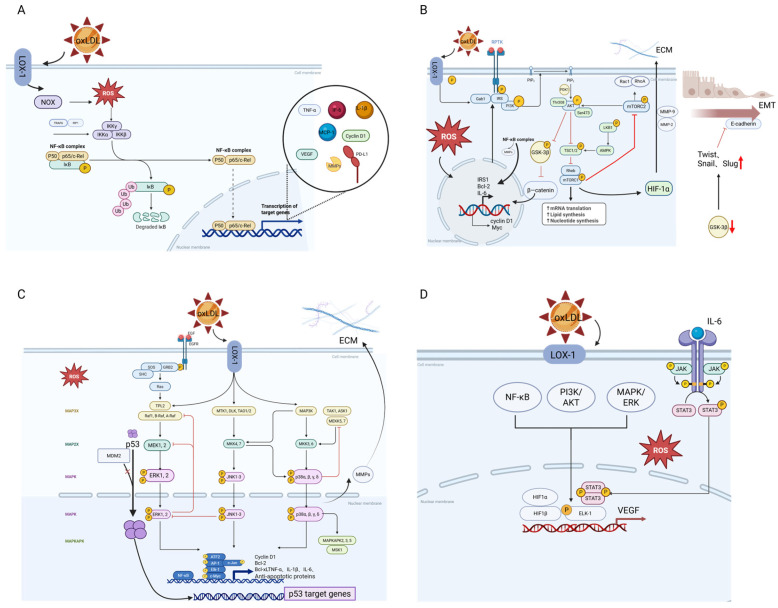
Expression pathways of oxLDL in CRC. (**A**) NF-κB Inflammatory Signaling: oxLDL binds to LOX-1, activating IKK and releasing NF-κB, which upregulates pro-inflammatory factors (e.g., TNF-α, IL-6) and immune checkpoint molecules (e.g., PD-L1), driving inflammation and immune evasion. (**B**) PI3K/Akt Signaling: oxLDL activates PI3K/Akt, enhancing cell survival, proliferation, and metabolic reprogramming by inhibiting apoptosis and upregulating anti-apoptotic genes (e.g., Bcl-2). Inactivation of GSK-3β enhances the expression of EMT-related factors (e.g., Twist, Snail) and suppresses the expression of the cell adhesion molecule E-cadherin, promoting cancer cell invasion and migration. (**C**) MAPK Signaling: oxLDL triggers ERK1/2, p38, and JNK pathways, promoting cell proliferation, invasion, and metastasis via transcription factors (e.g., AP-1). (**D**) VEGF signaling: oxLDL upregulates VEGF through NF-κB, PI3K/Akt, and MAPK, stimulating angiogenesis and immune evasion by enhancing endothelial cell proliferation and vascular permeability. Created in BioRender.com.

oxLDL is markedly overexpressed in CRC and is closely associated with tumor invasiveness, metastasis, and poor patient prognosis. oxLDL drives tumor progression by orchestrating inflammatory responses, metabolic reprogramming, and immune evasion within the tumor microenvironment. These effects are mediated through two interdependent mechanisms: (1) intrinsic expression dynamics of oxLDL and (2) activation of the LOX-1 receptor signaling pathway, as illustrated in Figure 3.

#### 2.1.1. Intrinsic Expression Dynamics of oxLDL

Elevated oxLDL levels, a hallmark of dysregulated lipid metabolism and chronic inflammation, are strongly associated with CRC pathogenesis. A Japanese cohort study demonstrated a significant positive correlation between plasma oxLDL levels and colorectal cancer risk [44,54]. In obese CRC patients, elevated oxLDL levels and heightened NF-κB immunoreactivity in tumor tissues were observed, with advanced-stage patients exhibiting even higher NF-κB activation (*p* < 0.05 for all comparisons). These findings implicate oxLDL-driven oxidative stress in promoting obesity-associated CRC via NF-κB signaling, suggesting its potential as a predictive and prognostic biomarker [55]. Furthermore, in high-fat diet-associated CRC, oxLDL activates macrophage LOX-1 receptors to induce CD206+ polarization, thereby upregulating stemness markers CD44 and CD133 in tumor cells [56]. Collectively, oxLDL emerges as a central node linking lipid dysregulation, inflammation, and tumor aggressiveness in CRC.

#### 2.1.2. LOX-1/NF-κB Inflammatory Signaling Pathway

Upon binding with oxLDL, the LOX-1 receptor activates the NF-κB signaling pathway, which plays a pivotal role in tumor initiation, progression, and immune evasion [57]. The activation of LOX-1 leads to the phosphorylation of the IκB kinase (IKK) complex, resulting in the degradation of IκB proteins and the subsequent release of NF-κB inhibition [58,59]. The activated NF-κB (p65/p50 complex) then translocates to the nucleus, initiating the transcription of genes associated with inflammation, immune regulation, and tumor metastasis [59].

NF-κB upregulates pro-inflammatory cytokines (such as TNF-α, IL-1β, IL-6, and IL-8) and chemokines (such as MCP-1 and CXCL1), exacerbating chronic inflammation within the TME [60]. This inflammatory milieu not only supports tumor cell proliferation and invasion but also promotes tumor cell survival by activating anti-apoptotic genes (such as Bcl-2 and IAPs) [61]. Additionally, NF-κB enhances the expression of immune checkpoint molecules (such as PD-L1 and CTLA-4), aiding tumor cells in evading immune surveillance [62]. Furthermore, NF-κB regulates the polarization of tumor-associated macrophages (TAMs), promoting the generation of M2-type macrophages, which further suppress anti-tumor immune responses [63]. Simultaneously, NF-κB induces the expression of matrix metalloproteinases (MMPs) and fibrinolytic enzymes, enhancing the invasive and metastatic capabilities of tumor cells [63,64]. Studies have shown that the oxLDL/LOX-1/NF-κB axis is activated to a significant extent in MSS CRC and is closely associated with tumor angiogenesis and distant metastasis [65].

#### 2.1.3. PI3K/Akt Signaling Pathway

oxLDL interacts with the lectin-like LOX-1, triggering the phosphatidylinositol 3-kinase/protein kinase B (PI3K/Akt) signaling cascade, which supports cancer cell proliferation, anti-apoptosis, invasion, metastasis, and metabolic reprogramming within the TME [66]. PI3K catalyzes the conversion of phosphatidylinositol 4,5-bisphosphate (PIP2) into phosphatidylinositol 3,4,5-trisphosphate (PIP3), leading to the recruitment and activation of Akt [67]. Activated Akt phosphorylates downstream targets, such as the mammalian target of rapamycin (mTOR) and glycogen synthase kinase-3β, promoting cancer cell proliferation, immune evasion, and metastasis. Akt activation inhibits apoptosis by phosphorylating pro-apoptotic proteins (e.g., BAD and BAX) and upregulating anti-apoptotic genes (e.g., Bcl-2), thereby protecting tumor cells from PCD [68]. Additionally, Akt regulates cell cycle proteins (e.g., Cyclin D1 and Cyclin E), facilitating the G1 to S phase transition and accelerating cell proliferation. The activation of the PI3K/Akt pathway also enhances inflammation within the TME by upregulating pro-inflammatory cytokines (e.g., IL-6 and TNF-α) and chemokines (e.g., MCP-1 and CXCL8) [69]. This inflammatory response supports tumor cell survival and proliferation while recruiting immunosuppressive cells, promoting immune evasion. Furthermore, the PI3K/Akt pathway increases the expression of MMPs, enhancing tumor cell migration and invasion [70]. Akt activation also regulates cytoskeletal reorganization-related molecules (e.g., focal adhesion kinase and β-catenin), increasing tumor cell motility and facilitating metastasis [71]. In summary, the oxLDL/LOX-1/PI3K/Akt axis is significantly activated in CRC and is closely associated with tumor metabolic reprogramming, such as enhanced glycolysis and fatty acid oxidation (FAO).

#### 2.1.4. MAPK Signaling Pathway

oxLDL interacts with LOX-1, leading to the activation of mitogen-activated protein kinase (MAPK) signaling pathways, including extracellular signal-regulated kinases 1 and 2 (ERK1/2), p38 MAPK, and c-Jun N-terminal kinase (JNK) [72]. These pathways regulate the expression of tumor-related genes, thereby enhancing the invasiveness and metastatic potential of CRC cells [73].

##### ERK1/2 Signaling Pathway

The ERK1/2 pathway is a classical branch of the MAPK Signaling cascade, playing a pivotal role in controlling cell proliferation, survival, differentiation, and metastasis [74]. The activation of ERK1/2 typically begins with signals from cell surface receptors, such as receptor tyrosine kinases, G-protein-coupled receptors, or integrins [75,76]. Upon the binding of oxLDL to LOX-1, conformational changes in LOX-1 activate downstream signaling molecules, including Src family kinases and Shc proteins, which subsequently recruit and activate the small GTPase Ras [76]. Activated Ras localizes to the plasma membrane, which engages and activates the Raf kinase. Raf then phosphorylates and activates MAPK/ERK kinase, which in turn phosphorylates ERK1/2 [77]. Once activated, ERK1/2 translocates to the nucleus, where it phosphorylates nuclear transcription factors such as ELK-1, c-Myc, and Fos, leading to the transcriptional upregulation of cell cycle proteins like Cyclin D1, thereby promoting cell cycle progression and proliferation [78]. Additionally, ERK1/2 activation results in the upregulation of anti-apoptotic proteins, including Bcl-2 and Bcl-xL, and can further activate the PI3K/Akt pathway, collectively contributing to the inhibition of apoptosis [79]. Moreover, ERK1/2 modulates the activity of cytoskeletal proteins, such as myosin and F-actin, enhancing the migratory and invasive capabilities of CRC cells [80].

##### p38 MAPK Signaling Pathway

The p38 MAPK pathway is a crucial branch of the MAPK family, primarily involved in stress responses, inflammation, cell differentiation, apoptosis, and immune regulation [81]. Upon the binding of oxLDL to the LOX-1 receptor, NOX is activated, leading to increased ROS levels, which subsequently activate the p38 MAPK pathway. This activation involves upstream kinases, such as TAK1 and MEKK3, initiating a MAPKKK-MAPKK cascade, ultimately resulting in the dual phosphorylation of p38 MAPK at conserved threonine (Thr180) and tyrosine (Tyr182) residues by MKK3 and MKK6 [82]. Activated p38 MAPK can translocate to the nucleus or remain in the cytoplasm to directly regulate target molecules. In the nucleus, p38 MAPK phosphorylates transcription factors such as ATF2, Elk-1, and NF-κB, thereby modulating the expression of cytokines, chemokines, and stress response-related genes [83]. Additionally, p38 MAPK can directly phosphorylate specific cytokines, protein kinases like MK2/3, and structural proteins, influencing various cellular functions [84]. Within the TME, p38 MAPK promotes the expression of pro-inflammatory factors like CXCL1 and CCL2, recruiting immunosuppressive cells such as TAMs [85]. This recruitment facilitates angiogenesis and immune evasion. Conversely, p38 MAPK can also induce the expression of pro-apoptotic genes like Bim and p53, triggering apoptosis in tumor cells and exhibiting tumor-suppressive effects [86].

##### JNK Signaling Pathway

JNK is a crucial member of the MAPK family, playing a pivotal role in regulating cell proliferation, apoptosis, migration, and immune responses [87]. Upon the binding of oxLDL to LOX-1, the upstream kinases of JNK, such as MKK4 and MKK7, are activated through ROS, leading to the phosphorylation and subsequent activation of JNK [88]. The activated JNK then translocates to the nucleus, where it enhances the transcriptional activity of the transcription factor c-Jun by phosphorylating it at Ser63 and Ser73 [89]. The phosphorylated c-Jun forms the AP-1 (activator protein-1) complex with c-Fos, which regulates the expression of a series of genes associated with tumor progression [90]. AP-1 promotes tumor cell invasion and metastasis by upregulating the expression of genes such as MMPs. Furthermore, JNK can enhance the motility and metastatic potential of tumor cells by regulating cytoskeletal reorganization and molecules related to epithelial–mesenchymal transition (EMT) [91].

#### 2.1.5. Angiogenesis Pathway

oxLDL significantly promotes angiogenesis in the TME by binding to the LOX-1 receptor and activating multiple signaling pathways, primarily through the regulation of the vascular endothelial growth factor (VEGF) signaling pathway. The VEGF signaling pathway is widely recognized as one of the key regulators of tumor angiogenesis [92]. oxLDL upregulates VEGF gene transcription and enhances its protein stability and secretion by activating the NF-κB, PI3K/AKT, and MAPK/ERK pathways [93]. Specifically, NF-κB dimers entering the nucleus can directly bind to the κB sites in the VEGF gene promoter region, thereby activating its transcription [94]. Simultaneously, activated ERK promotes the expression of VEGF and other angiogenesis-related genes by phosphorylating the transcription factor ELK-1 [95]. Additionally, oxLDL increases the production of ROS, which activates hypoxia-inducible factor 1α (HIF-1α). HIF-1α then forms a complex with HIF-1β and translocates to the nucleus, directly promoting VEGF gene transcription [96]. Furthermore, oxLDL enhances VEGF expression through autocrine or paracrine mechanisms by inducing the secretion of pro-inflammatory cytokines such as IL-6 and TNF-α. IL-6, as an indirect inducer of angiogenesis, exerts its pro-angiogenic activity by inducing VEGF [97]. TNF-α, on the other hand, upregulates the expression of AP-1 family members (e.g., c-Jun, c-Fos, and JunB) and enhances c-Jun phosphorylation levels through the JNK and AP-1-dependent pathways, thereby increasing VEGF mRNA and protein levels [98].

VEGF promotes the proliferation and migration of vascular endothelial cells by binding to its receptors and upregulates the expression of vascular endothelial cell junction proteins, such as VE-cadherin, thereby increasing vascular permeability and providing nutrients and oxygen to tumors [99]. Additionally, the newly formed vascular network facilitates the removal of metabolic waste, maintaining the stability of the TME for cancer cell growth. However, VEGF plays a complex dual role in the TME, not only promoting angiogenesis but also supporting tumor survival, invasion, and dissemination through immunosuppressive mechanisms [100]. Studies have shown that VEGF promotes the recruitment and expansion of myeloid-derived suppressor cells (MDSCs), and a strong correlation between VEGF and MDSCs has been observed in the TME. Infiltrating VEGFR2+ MDSCs inhibits the function of cytotoxic T lymphocytes (CTLs), thereby providing immune escape support for tumor cells [101]. Furthermore, VEGF inhibits the maturation of dendritic cells (DCs), keeping them in an immature state and reducing their antigen processing and presentation capabilities [102]. VEGFR2 is predominantly expressed in FOXP3+ regulatory T cells (Tregs), promoting their recruitment to tumor sites and suppressing the activation and proliferation of effector T cells, thereby enhancing tumor immunosuppression [103]. VEGF can also induce the increased expression of programmed death-1 (PD-1) on tumor-infiltrating CD8+ T cells through the VEGFR2-PLCγ-calcineurin-NFAT pathway, leading to T cell exhaustion [104]. These mechanisms collectively highlight the central role of VEGF in tumor angiogenesis and immune evasion, providing a theoretical foundation for anti-tumor therapies targeting the VEGF signaling pathway.

Furthermore, oxLDL, through its binding to the LOX-1 receptor, can also activate the downstream RAC1-GTPase signaling pathway, inducing the phosphorylation and activation of NOX. This leads to an increase in NADPH oxidation, resulting in the excessive generation of ROS [105]. The NOX family, which consists of seven members (NOX1 to NOX5, DUOX1, and DUOX2), plays a critical role in ROS signal transduction [106]. Compared to normal tissues, the mRNA expression of NOX1, NOX3, NOX4, DUOX1, and DUOX2 is significantly elevated in patients with MSS CRC [106]. Additionally, oxLDL can bind to the scavenger receptor CD36, activating the toll-like receptor 4 (TLR4)–MyD88–NOX signaling axis, further inducing excessive ROS production [107].

## 3. Mechanisms of Action and Resistance to ICIs in CRC

### 3.1. Clinical Applications and Limitations of ICIs in CRC

Cancer immunotherapy, which harnesses host immunity to achieve antitumor effects, has revolutionized oncology [108]. Immune checkpoint molecules such as PD-1/PD-L1 and CTLA-4 physiologically maintain immune tolerance by negatively regulating T cell activation [4]. ICIs, which block these pathways to restore immune balance in the TME, represent a transformative therapeutic strategy [4]. However, their clinical efficacy in CRC remains limited: approximately 85% of metastatic CRC (mCRC) cases exhibit MSS or mismatch repair-proficient phenotypes, characterized by low immunogenicity and insufficient effector T cell infiltration, resulting in objective response rates below 5% to ICI monotherapy [109]. Clinical trials underscore these challenges—pembrolizumab achieved 0% ORR in MSS patients, while regorafenib combined with nivolumab yielded modest ORR (7–33%) with pronounced efficacy heterogeneity in liver metastasis subgroups [110]. The resistance of MSS tumors to ICIs is attributed to a combination of immunosuppressive factor overexpression and neoantigen scarcity, collectively fostering an immunologically “cold” TME [111]. These findings underscore the urgent need to dissect the molecular basis of ICI resistance in MSS CRC and develop strategies to convert immunologically inert tumors into responsive ones.

### 3.2. Mechanisms of Immune Checkpoint Inhibitor Resistance in MSS Colorectal Cancer

From a molecular pathological perspective, CRC is broadly classified into two subtypes: (1) chromosomal instability, characterized by whole-chromosome or long-arm structural abnormalities, and (2) microsatellite instability (MSI), driven by high-frequency insertions/deletions in short tandem repeat regions [112]. The MSI phenotype primarily arises from deficient mismatch repair (dMMR) systems, which elevate tumor mutational burden (TMB) and generate abundant tumor-specific neoantigens, thereby activating robust T cell-mediated antitumor immunity [113]. In contrast, MSS CRC, marked by low TMB, scarce neoantigen epitopes, and persistently activated immunosuppressive pathways in the TME, exhibits intrinsic resistance to immune checkpoint inhibitors (ICIs) [113].

#### 3.2.1. Low Neoantigen Expression

Neoantigens—immunogenic peptides derived from somatic mutations—serve as tumor-specific antigens that activate cytotoxic T cells to eliminate malignant cells [114]. They originate from diverse mutational processes, including point mutations, deletions, and gene fusions [115]. Tumor cells process neoantigens into short peptide epitopes presented by MHC class I/II molecules for T cell recognition, initiating adaptive immune responses [116]. Notably, MSI CRC demonstrates a median neoantigen load ~20-fold higher than MSS tumors [117]. While MSI/dMMR tumors harbor a median of 121 neoantigens, the majority of MSS cases (excluding rare hypermutated subtypes) present fewer than 14 tumor-specific neoantigens [118]. This disparity is reflected in TMB metrics: MSS tumors average 4 mutations per megabase (mut/Mb), compared to 30 mut/Mb in MSI/dMMR tumors [119]. Such low TMB and neoantigen scarcity impair T cell priming and activation, underpinning the poor immunogenicity and ICI resistance of MSS CRC. The precise mechanisms by which neoantigen expression regulates T cell function remain incompletely defined, but potential pathways may involve (1) defective activation of naïve T cells due to insufficient neoantigen quantity, (2) T cell exhaustion induced by chronic exposure to suboptimal neoantigen levels, and (3) cross-tolerance triggered by limited epitope diversity [120,121,122].

#### 3.2.2. Immunosuppressive Tumor Microenvironment

The immunosuppressive TME of CRC critically regulates tumorigenesis and progression. Under physiological conditions, the immune system eliminates malignant cells through immunosurveillance, a concept first proposed by Marabelle et al. [123]. However, dysregulation of this defense mechanism enables tumor cells to evade immune destruction, facilitating uncontrolled proliferation and eventual clinical manifestation. During CRC progression, disrupted immune homeostasis drives aberrant immune cell functionality within the TME, establishing a profoundly immunosuppressive milieu [124]. Notably, MSS CRC exhibits distinct immunopathological features compared to MSI tumors, marked by differential immune cell infiltration across three functional compartments: tumor-infiltrating lymphocyte (TIL) zones, stromal regions, and the invasive front (tumor-lamina propria interface). The profound immunosuppression in MSS-CRC TME constitutes a core mechanism of immunotherapy resistance [125].

MSS-CRC TME is characterized by infiltration of immunosuppressive cell populations, including regulatory T cells (Tregs), tumor-associated macrophages (TAMs), myeloid-derived suppressor cells (MDSCs), and cancer-associated fibroblasts (CAFs) [126]. These cells cooperatively foster an immune-evasion-promoting niche through secretion of immunosuppressive cytokines (e.g., TGF-β, IL-10), functional exhaustion of effector T cells, and upregulation of immune checkpoint molecules [127]. Furthermore, the MSS-CRC TME harbors diverse malignant and stromal cell populations—such as neoplastic epithelial cells, mesenchymal cells, endothelial cells, and cancer stem cells—which interact with immunosuppressive cells to form a complex tumor ecosystem that exacerbates therapeutic challenges [128].

As pivotal immunomodulators within the TME, Tregs drive tumor immune evasion and progression via multifaceted mechanisms. While MSI tumors exhibit higher TIL density and CD8+ T cell infiltration, they paradoxically demonstrate elevated FOXP3 expression, reflecting increased Treg abundance compared to MSS tumors. This apparent contradiction likely stems from the necessity to counterbalance hyperactivated effector T cells in immunologically “hot” MSI tumors through proportional Treg expansion [129]. FoxP3+ Tregs establish a robust immunosuppressive network by overexpressing checkpoint molecules (CTLA-4, PD-1, LAG-3) and secreting inhibitory cytokines (IL-10, TGF-β, IL-35), thereby creating an immune-privileged niche for tumor survival. Emerging evidence implicates aberrant IL-33/ST2 signaling in Treg recruitment, functional activation, and TME remodeling, potentially underpinning Treg enrichment in MSS-CRC [129].

MSS and MSI colorectal cancers exhibit distinct myeloid cell compositions. MSI tumors develop unique immunological hubs within three key compartments: (1) tumor stroma, (2) myeloid cell-enriched regions (macrophages, dendritic cells, granulocytes), and (3) effector T cell zones expressing CXCR3 ligands at the tumor-lumen interface [71,72]. This cellular architecture demonstrates spatially organized precision—myeloid cells are strategically positioned to synergize with T cells, thereby optimizing tumor-cell interaction efficiency [130,131]. In contrast, MSS tumors exhibit constrained T cell-myeloid hub interactions due to deficient chemokine/cytokine signaling and altered adhesion molecule expression, reflecting the unique composition of their immunosuppressive TME.

Macrophages, characterized by remarkable phenotypic plasticity, polarize into two functionally divergent subsets: classically activated M1 and alternatively activated M2 macrophages [132]. M1 macrophages drive innate immune responses and antitumor immunity through pro-inflammatory cytokines (e.g., TNF-α, IL-6, IL-12) and cytotoxic mediators such as nitric oxide (NO) [133,134]. Conversely, M2 macrophages promote immunosuppression via IL-10, TGF-β, and arginase-1 (Arg-1) expression, facilitating tissue repair, angiogenesis, and tumor progression [133,134]. In the CRC microenvironment, TAMs predominantly adopt an M2-like phenotype [135], fostering malignancy through multifaceted mechanisms: secretion of matrix metalloproteinases (MMPs) and growth factors (e.g., VEGF), suppression of M1 macrophage antitumor activity, inhibition of T cell function via PD-L1 expression and arginine metabolism, and synergistic interactions with immunosuppressive cells (e.g., MDSCs, Tregs) [136].

MDSCs, a heterogeneous population of immature myeloid cells, play pivotal roles in tumor immune evasion by suppressing antitumor immunity and facilitating tumor progression [137]. Classified into monocytic (M-MDSCs) and polymorphonuclear (PMN-MDSCs) subsets based on morphological and surface marker profiles [138], MDSCs demonstrate elevated infiltration in peripheral blood and tumor tissues of MSS colorectal cancer patients compared to MSI cases, with their abundance correlating positively with disease progression and poor clinical outcomes [139]. Recruitment of MDSCs to the TME is primarily orchestrated by chemokines such as CCL2, secreted by tumor cells, stromal cells, and immune components within the TME. These chemokines not only guide MDSC migration but also amplify their immunosuppressive activity, fostering a pro-tumorigenic niche [140]. Functionally, MDSCs impair T cell-mediated antitumor immunity through three interconnected mechanisms: (1) direct secretion of ROS, NO, and Arg-1 to disrupt T cell activation and proliferation [141]; (2) induction of Tregs expansion and activation [142]; and (3) subversion of antigen-presenting cell function, thereby blunting tumor antigen-specific immune responses [143].

CAFs, critical non-immune stromal components of the TME, exert multifaceted regulatory roles in driving tumor malignancy [144]. Activated through sustained TGF-β signaling and molecular alterations such as p53 mutations, normal fibroblasts undergo phenotypic transformation into CAFs [145]. These activated CAFs participate in immune modulation via intricate cellular networks: they synergize with immunosuppressive cells (e.g., Tregs, TAMs, MDSCs) while directly suppressing cytotoxic T lymphocytes (CTLs) and natural killer (NK) cell functions [146]. Notably, in MSS CRC, CAF activation exhibits a strong positive correlation with TGF-β expression levels, underscoring their coordinated role in reinforcing the immunosuppressive TME [147].

Key cytokines within the TME, including IL-8 and TGF-β, drive the differentiation and expansion of tumor-associated neutrophils (TANs), which accelerate disease progression by stimulating angiogenesis and tumor growth [148]. TANs exert pro-tumorigenic effects primarily through the secretion of matrix metalloproteinase 9 (MMP9) to degrade extracellular matrix components and upregulation of VEGF, thereby enhancing tumor invasiveness and facilitating metastatic dissemination [149]. Notably, neutrophil extracellular traps (NETs)—DNA–protein scaffolds released by TANs—represent a critical mechanism in colorectal cancer progression [150]. These web-like structures not only provide physical support for tumor cell adhesion and migration but also establish an immune-privileged niche to shield cancer cells from immune surveillance. Furthermore, NETs enhance tumor cell metastatic potential by promoting EMT and modulating pre-metastatic niche formation.

#### 3.2.3. Tumor Angiogenesis

Tumor angiogenesis, a critical step in malignant progression, provides oxygen and nutrient supply to cancer cells through VEGF-mediated neovascularization. Beyond driving endothelial cell migration and vascular sprouting via VEGF/VEGFR signaling, VEGF serves as a key immunomodulator by fostering an immunosuppressive microenvironment. These immunosuppressive effects are mediated through multiple mechanisms: (1) VEGF/VEGFR signaling disrupts monocyte differentiation into dendritic cells (DCs), impairing antigen presentation [151]; (2) dual inhibition of T cell functionality—blocking lymphoid progenitor differentiation into CD4+/CD8+ effector T cells while suppressing immune cell infiltration into tumors via downregulation of endothelial adhesion molecules (e.g., ICAM-1, VCAM-1) [152]; (3) VEGF-A induces T cell exhaustion in MSS colorectal cancer by upregulating TOX expression [153]; and (4) enhanced recruitment of Tregs and MDSCs, coupled with M2 polarization of TAMs, further exacerbating the immunosuppressive milieu [154]. This multifaceted interplay positions VEGF not only as a driver of angiogenesis but also as a central orchestrator of immune evasion, highlighting its therapeutic relevance in overcoming resistance in MSS CRC.

#### 3.2.4. Tumor Metabolism

MSS-CRC exhibits significantly higher mitochondrial DNA (mtDNA) copy numbers and enhanced OXPHOS activity compared to MSI tumors [155]. While MSI CRC cells, under high TMB and immune pressure, predominantly utilize the Warburg effect (glycolysis-dominant metabolism) characterized by elevated lactate and reduced glucose levels, MSS CRC relies on OXPHOS to sustain energy metabolism, with mitochondrial functional integrity supporting malignant progression [156]. The TME of MSI tumors is defined by dynamic metabolic competition between cancer cells and T cells for glucose (a glycolysis substrate) and glutamine (a key glutaminolysis substrate). This competition drives T cells to metabolize glutamine into acetyl-CoA, fostering conditions conducive to immune cell infiltration, antigen presentation, and functional immune synapse formation [131]. Glutamine-derived acetyl-CoA supports T cell memory phenotype development and mitigates exhaustion, thereby enhancing antitumor immunity [157]. In contrast, MSS CRC demonstrates suppressed mtDNA levels in MSI tumors [158], with elevated mtDNA copy numbers stimulating OXPHOS to promote cell survival and metastasis [159]. Enhanced OXPHOS increases mitochondrial ROS (mtROS), inducing HIF-1α activation that upregulates PD-L1 expression, expands Tregs, and suppresses MHC-II-mediated antigen presentation [160]. Although MSS tumors exhibit lower lactate levels, OXPHOS-derived ATP is extruded via ENT1 channels, hydrolyzed to adenosine, and activates the CD73/A2AR pathway to inhibit CD8+ T cell cytotoxicity and amplify MDSCs [161,162].

### 3.3. Heterogeneity and Resistance Mechanisms in MSI CRC

While MSI-CRC is generally characterized by high TMB and robust immune infiltration, leading to favorable responses to ICIs, therapeutic resistance persists in a subset of patients due to molecular, epigenetic, and microenvironmental heterogeneity. Molecular subtyping, such as the Consensus Molecular Subtypes (CMSs), reveals divergent biological behaviors: CMS1 tumors exhibit CD8+ T cell-rich infiltrates and IFN-γ signaling, correlating with superior ICI efficacy, whereas CMS3 and CMS4 subtypes demonstrate stromal resistance mechanisms, including TGF-β pathway activation and extracellular matrix remodeling, which impair T cell trafficking and function [153,156]. Concurrent genetic alterations (e.g., *PIK3CA*, *PTEN*, or *BRAF* mutations) further exacerbate immunosuppression by activating oncogenic pathways (e.g., PI3K/Akt/mTOR) that upregulate PD-L1 or recruit MDSCs [53]. Epigenetic silencing of immune-related genes (e.g., HLA class I hypermethylation) and metabolic adaptations, such as elevated FAO and lactate secretion, suppress CTL activity while promoting Tregs expansion, enabling immune evasion despite high TMB [74,75]. Spatial heterogeneity in the TME, including CD8+ T cell exclusion from tumor cores and enrichment of immunosuppressive M2 macrophages or Tregs, is compounded by CAF-mediated stromal barriers (e.g., IL-6, VEGF), which physically and biochemically restrict T cell infiltration [66,67].

## 4. High oxLDL Expression in MSS CRC Drives ICI Resistance

**Figure 4:**
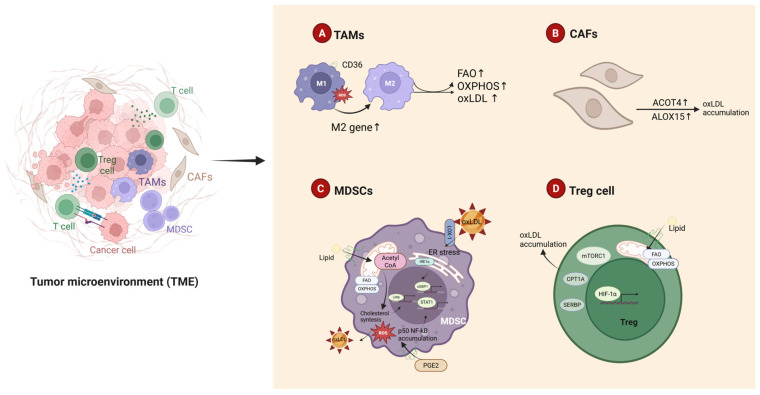
Differential accumulation of oxLDL in the immune microenvironment of MSS CRC. (**A**) TAMs: oxLDL is internalized by TAMs via LOX-1/CD36 receptors, enhancing FAO and OXPHOS, driving M2 polarization, and facilitating oxLDL uptake and intracellular accumulation. (**B**) CAFs: CAFs upregulate CD36 and LOX-1 expression through the secretion of IL-6 and TGF-β, promoting oxLDL uptake. ALOX15 and ACOT4 accelerate lipid peroxidation, leading to abnormal oxLDL accumulation. (**C**) MDSCs: MDSCs enhance FAO and OXPHOS, uptake oxLDL, and generate excessive ROS, inducing endoplasmic reticulum stress and suppressing T cell function. (**D**) Tregs: Tregs undergo lipid uptake and metabolic reprogramming mediated by CD36 and PPARγ, while the PD-1/CPT1A axis enhances FAO, further suppressing effector T cell activity. Collectively, these mechanisms contribute to the abnormal accumulation of oxLDL in the TME of MSS-type CRC, establishing a positive feedback loop that reinforces immune suppression and tumor progression. Created in BioRender.com.

**Figure 5:**
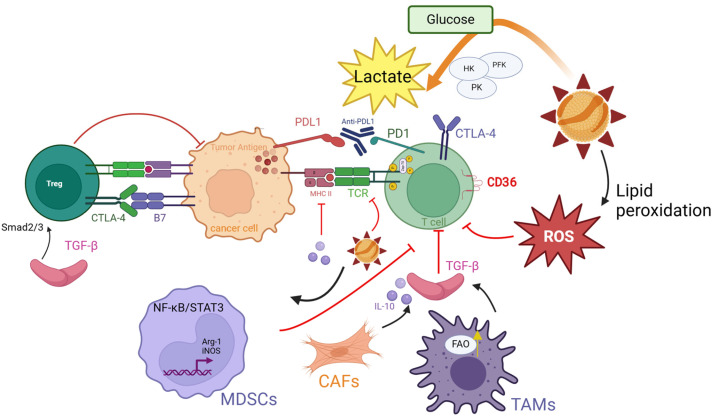
OxLDL-mediated resistance mechanisms to ICIs. (1) MDSCs: oxLDL activates the NF-κB/STAT3 axis in MDSCs, inducing overexpression of Arg-1 and iNOS, which deplete arginine and generate reactive nitrogen species, establishing a metabolic immunosuppressive barrier. (2) CAFs: oxLDL enhances CAF-mediated secretion of TGF-β and IL-10, fostering stromal fibrosis and physical barriers that impede T cell infiltration. CAFs further amplify immunosuppression by recruiting MDSCs and Tregs via chemokine signaling. (3) TAMs: oxLDL drives M2 polarization of TAMs through CD36/PPARγ signaling, enhancing their glycolytic flux and lactate production. Lactate activates the MCT1/NF-κB/COX-2 axis to upregulate PD-L1 on neutrophils and PD-1 on Tregs, while TME acidification directly suppresses cytotoxic T cell activity. (4) Metabolic Reprogramming Synergy: oxLDL upregulates glycolysis enzymes (e.g., HK, PKM) in tumor cells and TAMs, depleting glucose and inducing T cell energy exhaustion. Concurrently, oxLDL activates FAO via CD36/CPT1A, fueling immunosuppressive functions of TAMs and MDSCs. Created in BioRender.com.

The distinct molecular landscapes of MSS and MSI colorectal cancers—including differential TMB, immunosuppressive TME features, and metabolic reprogramming—not only underlie the limited efficacy of ICIs in MSS-CRC but also drive the subtype-specific elevation of oxLDL levels, with MSS tumors exhibiting significantly higher oxLDL expression than MSI counterparts, as illustrated in Figure 4. Accumulating evidence from breast cancer, melanoma, and ovarian cancer studies demonstrates that oxLDL contributes to immune evasion and therapy resistance across malignancies. In MSS CRC, aberrant oxLDL accumulation exacerbates immunosuppression through a multi-dimensional network: it modulates TMB-associated neoantigen presentation, impairs immune cell effector functions (e.g., cytotoxic T cell activity and dendritic cell maturation), and rewires metabolic pathways to sustain an immunosuppressive niche (Figure 5). This synergistic interplay between oxLDL-driven TME remodeling and ICI resistance highlights oxLDL as a therapeutic target for overcoming immunotherapy limitations in MSS CRC.

### 4.1. Low TMB in MSS CRC Drives oxLDL Accumulation

In MSI colorectal cancer, neoantigens presented via the major histocompatibility complex (MHC) suppress tumor proliferation and reduce oxidative stress, thereby inhibiting ROS-mediated lipid oxidation and oxLDL generation [163]. In contrast, MSS-CRC, characterized by low TMB and neoantigen scarcity, sustains oxidative stress through LOX-1/NF-κB pathway activation, leading to progressive oxLDL accumulation in the immunosuppressive microenvironment. Mechanistically, approximately 53.9% of MSS patients harbor KRAS mutations, which drive sustained RAF/MEK/ERK signaling to upregulate NOX expression, triggering ROS overproduction [164,165]. ROS not only directly oxidize LDL to form oxLDL but also enhance OXPHOS via PI3K/Akt/mTOR activation, establishing a self-reinforcing “ROS-oxLDL-OXPHOS” feedforward loop [166]. oxLDL exacerbates genomic instability by inducing oxidative DNA damage (elevated 8-OHdG levels), which disrupts DNA replication/transcription fidelity and generates non-functional or synonymous mutations (e.g., in non-coding regions or silent amino acid changes). These mutations fail to produce immunogenic neoantigens, resulting in diminished CD8+ T cell recognition and a “cold tumor” phenotype. Furthermore, oxLDL activates NF-κB signaling to overexpress DNA methyltransferases, causing hypermethylation of the MSH2/MLH1 promoter regions and subsequent transcriptional silencing [167,168], thereby compounding immune evasion mechanisms in MSS-CRC.

### 4.2. oxLDL Accumulation in MSS CRC Immunosuppressive Microenvironment Drives Therapy Resistance

The TME of MSS-CRC is characterized by low immune infiltration and heightened immunosuppression. The differential accumulation of oxLDL in MSS-CRC TME exacerbates immune evasion through multidimensional mechanisms, ultimately conferring resistance to ICIs.

In MSS-CRC, oxLDL enters cells via low-density lipoprotein receptor-mediated endocytosis, where lysosomal acid lipase hydrolyzes it into free fatty acids and cholesterol, driving dysregulated lipid metabolism [169]. The MSS TME exhibits M2-polarized macrophages with enhanced FAO and OXPHOS [170,171]. Mechanistically, deficiency of fatty acid-binding protein 5 causes intracellular accumulation of long-chain unsaturated fatty acids in macrophages, which sustain FAO and OXPHOS via peroxisome proliferator-activated receptor δ activation [172]. MSS TAMs overexpress lipid transporters CD36 and cholesterol efflux pumps, promoting oxLDL uptake and intracellular retention [173]. Single-cell transcriptomics reveals that CD36+ TAMs exhibit upregulated expression of FAO enzymes [carnitine palmitoyltransferase 1A (CPT1A), Acyl-CoA Dehydrogenase Very Long Chain (ACADVL)] and enhanced mitochondrial respiratory chain activity, leading to ROS overproduction [174]. Excessive ROS activate JAK1 phosphorylation and inhibit SHP1 dephosphorylation, driving persistent STAT6 activation, which further elevates TME oxLDL levels. oxLDL reciprocally activates NF-κB and PI3K/Akt pathways via CD36/LOX-1 receptors, driving M2 polarization of TAMs and establishing a self-reinforcing “oxLDL accumulation-immunosuppression” feedback loop. Under oxLDL stimulation, TAMs upregulate TGF-β and IL-10 secretion via TLR4/LOX-1-mediated STAT3 phosphorylation [175]. These immunosuppressive factors suppress antitumor immunity through dual mechanisms: (1) TGF-β promotes naïve T cell differentiation into regulatory T cells (Tregs) and enhances their suppressive activity via Smad2/3 signaling [176]; (2) IL-10 diminishes effector T cell activation by suppressing MHC-II and co-stimulatory molecules (CD80/CD86) on antigen-presenting cells [177]. Notably, TGF-β directly impairs CD8+ T cell cytotoxicity by downregulating granzyme B and perforin expression—an effect exacerbated during anti-PD-1/PD-L1 therapy, contributing to reduced ICI response rates [178].

CAFs dynamically regulate the lipid metabolic network, creating a vicious cycle that promotes the abnormal accumulation of oxLDL in the TME. CAFs highly express fatty acid-binding protein 4 (FABP4), which specifically binds long-chain fatty acids and enhances their transmembrane transport, significantly increasing lipid accumulation in tumor and stromal cells. This provides a rich substrate pool for the oxidative modification of LDL [179]. This lipid overload state accelerates lipid peroxidation chain reactions by activating lipoxygenase and cyclooxygenase pathways, thereby promoting oxLDL generation. Additionally, CAFs-secreted IL-6 and TGF-β regulate oxLDL metabolism through dual signaling axes: IL-6 induces the transcriptional activation of the scavenger receptor CD36 via the JAK/STAT3 pathway, enhancing the endocytic efficiency of oxLDL in tumor cells [180]; meanwhile, TGF-β synergistically activates NF-κB signaling through a Smad-dependent mechanism, upregulating the expression of the LOX-1 receptor and establishing a bidirectional positive feedback loop between ligand and receptor [181].

Notably, CAFs further amplify the pathological effects of oxLDL by constructing a unique lipid metabolic enzyme network. Acyl-coenzyme A thioesterase 4 (ACOT4) catalyzes the hydrolysis of fatty acyl-CoA, releasing free fatty acids as precursors for lipid oxidation [182]. Simultaneously, lipoxygenase ALOX15 oxidizes arachidonic acid to generate pro-inflammatory mediators, synergistically promoting lipid peroxidation [183]. The coordinated action of the ACOT4–ALOX15 metabolic axis not only drives the abnormal accumulation of oxLDL but also remodels the metabolic landscape of the TME. This induces the excessive secretion of VEGF and upregulates ATP synthase expression, simultaneously meeting the malignant demands of tumor cells for immune evasion, angiogenesis, and energy metabolism [184]. Ultimately, CAF-driven lipid metabolic reprogramming establishes a self-sustaining pathological ecosystem, providing multidimensional metabolic support for progression.

The functional realization of MDSCs in the TME relies on critical metabolic reprogramming to meet their high energy demands and sustain immunosuppressive and pro-tumor activities. Studies have shown that tumor-infiltrating MDSCs switch their primary energy source from glycolysis to lipid metabolism by enhancing FAO and OXPHOS, thereby supporting their long-term survival and functional performance [185]. In the TME, MDSCs drive the abnormal accumulation of oxLDL through multidimensional mechanisms: at the metabolic level, the enhancement of FAO/OXPHOS leads to a burst of mitochondrial ROS generation, synergizing with inflammatory mediators such as prostaglandin E2 to exacerbate lipid peroxidation and promote oxLDL formation [186]; at the receptor level, MDSCs highly express scavenger receptors CD36 and LOX-1, significantly improving the uptake efficiency of oxLDL; at the signaling level, the tumor-derived granulocyte–macrophage colony-stimulating factor induces the upregulation of fatty acid transport protein 2 in MDSCs by activating the STAT3 signaling pathway, thereby enhancing lipid uptake and oxidative modification, forming a vicious cycle of “lipid overload–ROS elevation” [187].

The arachidonic acid ingested by MDSCs is converted into PGE2 through the cyclooxygenase pathway, which drives the nuclear translocation of the NF-κB p50 subunit, promoting the expression of NO synthase. This subsequently inhibits effector T cell function through NO-mediated immunosuppressive mechanisms [188]. In addition, calcium homeostasis imbalance and lipid metabolism disorders in the TME can induce endoplasmic reticulum stress, activating the unfolded protein response and enhancing the expression of lipid synthesis-related enzymes through the PERK-eIF2α pathway, further exacerbating the pathological accumulation of oxLDL [188]. Notably, CAFs polarize monocytes into MDSCs by secreting IL-6 and TGF-β, while oxLDL itself can act as an immunomodulatory molecule. By binding to TLR4 on the surface of MDSCs, oxLDL activates the NF-κB and MAPK pathways, inducing the expression of immunosuppressive cytokines (e.g., IL-10 and TGF-β) and forming an “oxLDL–TLR4–immunosuppression” cascade amplification effect [189]. Mechanistically, oxLDL activates the NF-κB/STAT3 signaling axis in MDSCs, driving overexpression of Arg-1 and inducible nitric oxide synthase (iNOS) to establish a metabolic immunosuppressive barrier that depletes arginine and generates cytotoxic reactive nitrogen species [190]. Concurrently, oxLDL-derived ROS upregulate chemokines CXCL1/CCL2, recruiting MDSCs from the bone marrow to the tumor site and forming a self-reinforcing “ROS-chemokine-MDSC infiltration” feedback loop. This multilayered immunometabolic regulatory network culminates in systemic suppression of effector T cell functionality within the TME, creating a microenvironmental foundation for resistance to immune checkpoint inhibitors.

In the TME of MSS, Tregs exhibit a significant proliferative advantage over Teffs, with their metabolic characteristics closely linked to their immunosuppressive functions. Single-cell metabolomic analyses have revealed that Tregs maintain energy homeostasis by enhancing OXPHOS, FAO, and cholesterol synthesis pathways. This is characterized by the upregulation of lipid uptake-related molecules, such as CD36 and fatty acid synthase (FASN), alongside the downregulation of glucose transporter GLUT1 [191,192]. This metabolic reprogramming enables Tregs to efficiently utilize oxLDL as an energy substrate while reducing nutrient competition with Teffs through the suppression of glycolysis. Mechanistic studies have shown that the aberrant activation of peroxisome proliferator-activated receptor gamma (PPARγ) in Tregs is a central driver of their metabolic adaptation [193]. PPARγ directly binds to the promoter regions of CD36 and FABP4, upregulating their mRNA expression levels and significantly enhancing the cellular capacity for oxLDL uptake [194]. The ingested oxLDL is degraded in lysosomes, releasing free fatty acids that enter the FAO cycle via CPT1A-mediated mitochondrial membrane transport, ultimately generating substantial ATP to support the long-term survival of Tregs. In stark contrast, pro-inflammatory immune cells (e.g., Teffs, Th17 cells, and M1-type macrophages) primarily rely on glycolysis for rapid ATP production. While this metabolic mode meets the energy demands required for their expansion and inflammatory cytokine secretion, it leads to lactate accumulation and acidification in the TME, further suppressing the function of effector immune cells. Cheng et al. found that the activity of sterol regulatory element-binding proteins (SREBPs) is significantly upregulated in tumor-infiltrating Tregs. The downstream regulator SREBP cleavage-activating protein drives the transcriptional activation of genes related to cholesterol and fatty acid synthesis by mediating the proteolytic processing of SREBPs [195]. This metabolic reprogramming directly promotes the biosynthesis of oxLDL, thereby supporting the specific expansion of OX40+ Tregs in the TME of models and hepatocellular carcinoma patients. OX40 (also known as the TNF receptor superfamily member 4, TNFRSF4, or CD134) shapes the metabolic phenotype of Tregs through a dual mechanism: on the one hand, it remodels the lipid composition of the cell membrane by activating the FAS signaling pathway [196]; on the other hand, it enhances glycolytic flux to provide biosynthetic precursors, collectively promoting the proliferation and immunosuppressive functions of Tregs [197]. Notably, the high expression of PD-1 in Tregs upregulates the protein level of CPT1A, inducing the FAO of endogenous lipids and driving the excessive generation of mitochondrial acetyl-CoA, thereby exacerbating the pathological accumulation of oxLDL [198]. Additionally, the mechanistic target of rapamycin complex 1 (mTORC1) signaling pathway promotes the metabolic flux of the mevalonate pathway by regulating the rate-limiting enzymes of cholesterol biosynthesis (e.g., HMGCR), which is crucial for maintaining the immunosuppressive activity of Tregs [199]. Mechanistic studies have shown that Raptor, an effector protein of mTORC1, synergistically activates the SREBP2 and LXRα signaling axes, forming a “cholesterol synthesis–reverse transport” dynamic balance network, ultimately endowing Tregs with the ability to maintain metabolic adaptability in the nutrient-deprived TME [200]. These findings reveal the deep interaction between the lipid metabolic network and immune checkpoint molecules, providing new intervention targets for reversing immunosuppression by targeting metabolic reprogramming.

Furthermore, the N2 pro-tumor phenotype of tumor-associated neutrophils (TANs) also exhibits unique metabolic characteristics. This metabolic reprogramming supports their immunosuppressive functions through lipid dynamic imbalance and energy metabolism remodeling [201]. In N2 neutrophils, the expression of lipid uptake-related genes (e.g., CD36, FABP4) and key regulators of lipid droplet (LD) formation (e.g., PLIN2) is significantly upregulated, while the expression of lipid droplet degradation enzymes (e.g., ATGL) and rate-limiting enzymes of fatty acid β-oxidation (e.g., CPT1A) is suppressed, leading to the abnormal accumulation of intracellular oxLDL and lipid droplets [202,203].

### 4.3. Synergistic Crosstalk Between Metabolic Reprogramming and Immunosuppressive Microenvironment

In MSS-CRC, OXPHOS activity drives ROS overproduction, which directly promotes oxLDL accumulation via LDL oxidation. Concurrently, TGF-β signaling in MSS CRC amplifies ROS generation while depleting glutathione (GSH) levels, further compromising ROS detoxification capacity [204]. In contrast, high TMB in MSI tumors recruits immune cells that secrete interferon-γ (IFN-γ), activating the JAK/STAT pathway to suppress mitochondrial ROS production and mitigate oxLDL accumulation [205].

OxLDL critically mediates ICI resistance by reshaping the metabolic landscape of the TME and orchestrating crosstalk between glucose metabolism and immune responses. Binding to the CD36 receptor on tumor cells, oxLDL activates the PI3K/AKT/mTOR pathway, upregulating key glycolytic enzymes (e.g., hexokinase, phosphofructokinase, pyruvate kinase) while stabilizing HIF-1α to enhance glycolysis in both tumor cells and M2-polarized TAMs [206]. This metabolic reprogramming suppresses antitumor immunity through three interconnected mechanisms: First, competitive glucose uptake by tumor cells and TAMs induces nutrient deprivation in TILs, suppressing mTOR activity and reducing effector cytokine secretion (e.g., IFN-γ, TNF-α) [207]. Second, glycolytic flux elevates lactate production, which activates the MCT1/NF-κB/COX-2 axis to upregulate PD-L1 in neutrophils and drives NFAT1 nuclear translocation to increase PD-1 expression in Tregs, reinforcing an immunosuppressive niche [208]. Finally, lactate-induced TME acidification directly impairs CTL activity while promoting M2 polarization of TAMs, further amplifying immune evasion [209].

OxLDL drives metabolic heterogeneity across TME components to sustain immunosuppression: in tumor cells, it activates the SREBP1/FASN axis to enhance fatty acid synthesis, supplying acetyl-CoA precursors for glycolysis [210,211,212]; in TAMs, it triggers PPARγ/LXRα signaling to promote cholesterol reverse transport and lipid droplet formation; and in Tregs, it upregulates CPT1A to fuel FAO, thereby energizing their immunosuppressive functions. This metabolic divergence fosters competitive nutrient depletion within the TME, establishing a self-reinforcing “metabolic deprivation-immunosuppression” cycle that underlies resistance to ICIs. Collectively, these mechanisms underscore the therapeutic imperative to develop combinatorial strategies targeting oxLDL-mediated pathways to disrupt immunosuppressive metabolic reprogramming and restore antitumor immunity.

## 5. Potential Therapeutic Strategies

Therapeutic strategies targeting oxLDL-related signaling pathways have demonstrated significant anti-metastatic potential in various in situ cancer models, including melanoma, oral cancer, breast cancer, and ovarian cancer [213]. Current intervention strategies for oxLDL primarily focus on blocking its interaction with receptors and regulating OS levels. In terms of receptor blockade, the E06 monoclonal antibody specifically binds to the oxidized phospholipid epitope of oxLDL, effectively inhibiting the activation of the oxLDL–TLR4/CD36 signaling axis. This reduces the expansion of Tregs, the activation of MDSCs, and the secretion of immunosuppressive factors such as IL-10 and TGF-β. Small-molecule inhibitors like Sulfosuccinimidyl oleate and Berberine target CD36 and LOX-1 receptors, respectively, blocking the endocytosis of oxLDL and its downstream pro-inflammatory signal transduction. These inhibitors have shown promising effects in reshaping the immune microenvironment in preclinical studies [214].

In terms of OS regulation, antioxidants indirectly reduce oxLDL levels by decreasing the oxidative modification of LDL, thereby creating favorable conditions for restoring the metabolic functions of immune cells. N-acetylcysteine significantly reduces ROS levels in the TME by elevating GSH levels, thereby inhibiting oxLDL generation [215]. Polyphenolic compounds exert their effects through a dual mechanism: not only do they directly scavenge free radicals to inhibit lipid peroxidation, but they also enhance tumor cell sensitivity to treatment by regulating DNA repair processes. Additionally, antioxidant vitamins such as vitamin E and vitamin C inhibit lipid peroxidation by directly scavenging free radicals, showing favorable therapeutic effects in clinical studies on CRC [216].

Furthermore, the abnormal proliferation of tumor cells is highly dependent on the continuous activation of lipid synthesis pathways. This metabolic reprogramming not only provides the lipid precursors required for biomembrane construction but also supports their malignant phenotype by maintaining cell membrane stability. Lipid metabolism disorder is a key driver of oxLDL generation. Therefore, targeting lipid metabolism pathways can effectively reduce oxLDL levels and its mediated immunosuppressive effects. Statins suppress HMG-CoA reductase activity to block the mevalonate pathway, significantly reducing intracellular cholesterol levels and, thereby, limiting LDL oxidation and oxLDL generation. Beyond their lipid-lowering effects, statins enhance immunotherapy sensitivity by alleviating T cell exhaustion and reducing immunosuppressive cell infiltration (e.g., Tregs, MDSCs), ultimately lowering cancer-related mortality and tumor recurrence rates [217]. Additionally, drugs targeting FAO, such as etomoxir, inhibit the activity of CPT1, preventing long-chain fatty acids from entering mitochondria for β-oxidation. This weakens the energy supply of tumor cells and immunosuppressive cells (e.g., TAMs and MDSCs), indirectly reducing the accumulation of oxLDL in the TME and its maintenance of immunosuppressive phenotypes [218].

**Figure 6:**
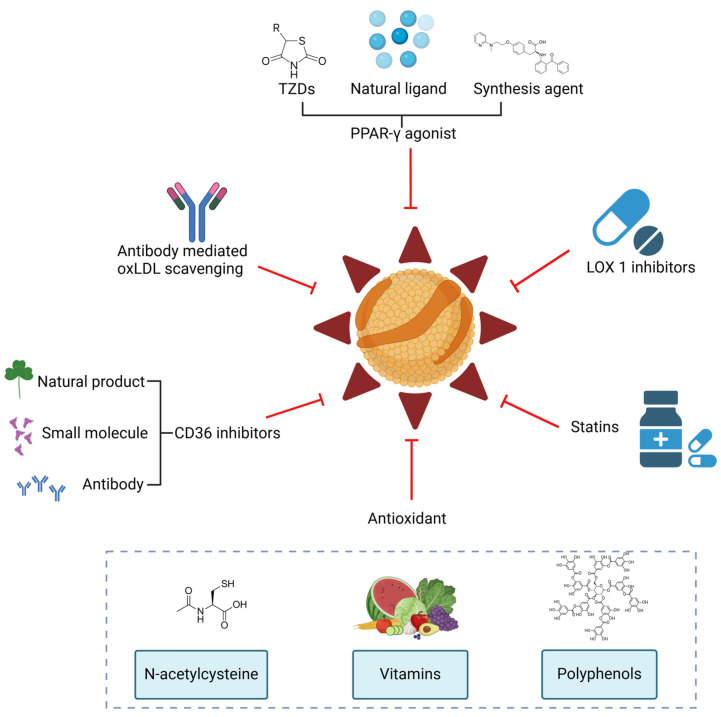
Potential therapeutic strategies targeting oxLDL-mediated immunosuppression. (1) Antibody-mediated oxLDL scavenging: The E06 monoclonal antibody specifically binds to oxidized phospholipid epitopes on oxLDL, inhibiting the oxLDL-TLR4/CD36 signaling axis. (2) LOX-1 and (3) CD36 Inhibitors: These agents block oxLDL internalization and downstream signaling. (4) Antioxidants: N-acetylcysteine and polyphenolic compounds scavenge ROS, reduce oxLDL generation, and restore immune cell function. (5) Metabolic Modulation: Statins inhibit HMG-CoA reductase, lowering cholesterol synthesis and oxLDL levels. (6) PPAR-γ Agonists: These agents upregulate ABCA1/ABCG1 to promote cholesterol reverse transport while suppressing the NF-κB pathway, alleviating inflammation and immune suppression. These multi-target strategies hold promise for reversing oxLDL-mediated immune suppression and enhancing ICI efficacy. Created in BioRender.com.

PPAR-γ, a nuclear receptor transcription factor, plays a central role in lipid metabolism and inflammation regulation. PPAR-γ agonists (e.g., rosiglitazone) inhibit the pathological effects of oxLDL through a dual mechanism: on the one hand, they promote reverse cholesterol transport by upregulating the expression of ABCA1 and ABCG1, thus reducing lipid accumulation; on the other hand, they suppress the NF-κB signaling pathway to decrease the expression of pro-inflammatory factors such as IL-6 and TNF-α while inducing the secretion of anti-inflammatory mediators (e.g., IL-10) [219]. Notably, PPAR-γ agonists can also regulate macrophage polarization, reducing the proportion of M2-type TAMs, thereby alleviating oxLDL-mediated lipid metabolic stress [220]. The combined use of PPAR-γ agonists and antioxidants (e.g., N-acetylcysteine) synergistically enhances antioxidant defense capabilities. By increasing GSH levels, this combination reduces ROS-mediated lipid peroxidation, ultimately reshaping the metabolic–immune balance in the TME. This multi-target combination strategy provides a new direction for reversing oxLDL-related immunosuppression and overcoming treatment resistance (Figure 6).

Recent clinical efforts to target oxLDL-related pathways are summarized in Table 1. While direct oxLDL depletion strategies remain largely preclinical, indirect approaches—such as statins, antioxidants, and efatutazone—have entered early-phase trials in cancer. For example, statins (e.g., atorvastatin) are being evaluated for their chemopreventive and immunomodulatory effects in CRC, with secondary outcomes including oxLDL reduction. Antioxidants like Curcumin have shown promise in reducing oxidative stress biomarkers (e.g., oxLDL, 8-OHdG) in CRC patients, though their efficacy as monotherapy is limited. These trials underscore the potential of oxLDL-targeted therapies but also highlight the need for larger, biomarker-driven studies to validate their role in overcoming ICI resistance.

## 6. Limitations and Future Directions

Although strategies targeting the clearance of oxLDL provide new avenues for overcoming resistance to ICIs in MSS CRC, current research still faces several limitations. First, while oxLDL levels are significantly correlated with CRC progression and the formation of an immunosuppressive microenvironment, its utility as a standalone diagnostic biomarker remains limited, likely due to interindividual heterogeneity in cancer immune responses [228]. Therefore, future studies should explore combining oxLDL with other immune or metabolic markers (e.g., PD-L1 expression, TMB, lactate levels) to improve the sensitivity and specificity of CRC diagnosis and provide a more comprehensive molecular classification for personalized therapy.

The clinical translation of oxLDL clearance strategies requires several critical scientific challenges to be addressed. First, the molecular mechanisms underlying oxLDL interactions with its receptors (e.g., CD36, LOX-1) need further elucidation to develop more selective and efficient targeted drugs. For instance, small-molecule inhibitors or antibody drugs designed based on structural biology may precisely block oxLDL-receptor binding, minimizing off-target effects and enhancing therapeutic safety. Second, the design of combination therapies must systematically evaluate drug dosage, timing, and synergistic mechanisms. Additionally, in-depth studies leveraging metabolomics and single-cell sequencing technologies may uncover novel details of oxLDL-regulated immunometabolic networks, offering theoretical support for innovative combination strategies. Finally, personalized treatment approaches for MSS CRC patients should integrate genomic profiles, metabolic phenotypes, and immune microenvironment status to achieve precision medicine goals. While this review focuses on CRC, the conserved role of oxLDL in other malignancies—such as its association with VEGF-driven angiogenesis in ovarian cancer and TAM polarization in lung cancer—underscores its broader therapeutic relevance. Future studies should explore the extrapolation of oxLDL-targeted strategies developed for CRC to other oxidative stress-rich tumors, while multidisciplinary approaches integrating technological innovations (e.g., metabolomic profiling, nanomedicine) may optimize oxLDL-directed therapies to achieve enhanced clinical benefits for MSS CRC patients.

## Figures and Tables

**Table 1:** Clinical trials and studies targeting oxLDL in CRC.

Intervention	Target/Mechanism	Phase	Population	Key Findings/Outcomes	Identifier/Reference
Atorvastatin	HMG-CoA reductase inhibitor	II	Patients with colorectal cancer or multiple/advanced colorectal adenomas	Reduced CRC risk	Limburg, P.J et al. [221]
Simvastatin	HMG-CoA reductase inhibitor	II	Patients with locally advanced rectal cancer	No improvement in pathological complete response, though no increased toxicity observed	Jo, H et al. [222]
Simvastatin	HMG-CoA reductase inhibitor	II	KRAS-mutant CRC patients refractory to irinotecan/oxaliplatin chemotherapy	Demonstrated promising efficacy and safety	Lee, J et al. [223]
Curcumin	Antioxidant	I	Patients with advanced CRC refractory to standard chemotherapy	Exhibited anticancer activity via GST activation, PGE2 suppression, and inhibition of oxidative DNA adduct (M1G) formation	Sharma, R.A et al. [224]
Vitamin C	Antioxidant	III	mCRC patients (*n* = 442) with normal G6PD status, no prior metastatic treatment	High-dose vitamin C + chemotherapy failed to improve PFS vs. chemotherapy alone but may benefit RAS-mutant mCRC patients	Wang, F et al. [225]
Vitamin E	Antioxidant	I	Advanced CRC patients (Dukes C/D stage)	Suppressed ROS production and enhanced NK cell function in CRC patients	Hanson, M.G.V et al. [226]
Efatutazone	PPARγ agonist	I	Japanese patients with metastatic CRC (mCRC)	Efatutazone + FOLFIRI showed acceptable safety and evidence of disease stabilization in mCRC	Komatsu, Y. et al. [227]

## Data Availability

Not applicable.

## Abbreviations

The following abbreviations are used in this manuscript:

OS	Oxidative stress
ROS	Reactive oxygen species
LDL	Low-density lipoprotein
oxLDL	Oxidized low-density lipoprotein
CRC	Colorectal cancer
PCD	Programmed cell death
ETC	Electron transport chain
H_2_O_2_	Hydrogen peroxide
O_2_^−^	Superoxide anions
NOX	NADPH oxidase
NOS	Nitric oxide synthase
COX	Cyclooxygenases
LOX	Lipoxygenases
MDA	Malondialdehyde
4-HNE	4-hydroxy-2-nonenal
LOX-1	Oxidized LDL receptor-1
TAMs	Tumor-associated macrophages
MMPs	Matrix metalloproteinases
PI3K/Akt	Phosphatidylinositol 3-kinase/protein kinase B
PIP2	Phosphatidylinositol 4,5-bisphosphate
PIP3	Phosphatidylinositol 3,4,5-trisphosphate
mTOR	Mammalian target of rapamycin
MAPK	Mitogen-activated protein kinase
ERK1/2	Extracellular signal-regulated kinases 1 and 2
JNK	c-Jun N-terminal kinase
VEGF	Vascular endothelial growth factor
HIF-1α	Hypoxia-inducible factor 1α
TME	Tumor microenvironment
MDSCs	Myeloid-derived suppressor cells
DCs	Dendritic cells
Tregs	Regulatory T cells
TLR4	Toll-like receptor 4
MSI	Microsatellite instability-high
TMB	Tumor mutation burden
OXPHOS	Oxidative phosphorylation
GSH	Glutathione
MHC	Major histocompatibility complex
FAO	Fatty acid oxidation
CAFs	Cancer-associated fibroblasts
FABP4	Fatty acid-binding protein 4
ACOT4	Acyl-coenzyme A thioesterase 4
FASN	Fatty acid synthase
PPARγ	Peroxisome proliferator-activated receptor gamma
SREBPs	Sterol regulatory element-binding proteins
CPT1A	Carnitine palmitoyltransferase 1A
mTORC1	Mechanistic target of rapamycin complex 1
TANs	Tumor-associated neutrophils
ICIs	Immune checkpoint inhibitors
TCR	T-cell receptor
TGF-β	Transforming growth factor-beta
Arg1	Arginase-1
TILs	Tumor-infiltrating lymphocytes
MPO	Myeloperoxidase
EMT	Epithelial–mesenchymal transition
dMMR	Deficient mismatch repair
CTL	Cytotoxic T lymphocyte

## References

[1] Perillo B., Di Donato M., Pezone A., Di Zazzo E., Giovannelli P., Galasso G., Castoria G., Migliaccio A. (2020). ROS in cancer therapy: The bright side of the moon. Exp. Mol. Med..

[2] Snezhkina A.V., Kudryavtseva A.V., Kardymon O.L., Savvateeva M.V., Melnikova N.V., Krasnov G.S., Dmitriev A.A. (2019). ROS Generation and Antioxidant Defense Systems in Normal and Malignant Cells. Oxid. Med. Cell Longev..

[3] Foksinski M., Rozalski R., Guz J., Ruszkowska B., Sztukowska P., Piwowarski M., Klungland A., Olinski R. (2004). Urinary excretion of DNA repair products correlates with metabolic rates as well as with maximum life spans of different mammalian species. Free Radic. Biol. Med..

[4] Wei S.C., Duffy C.R., Allison J.P. (2018). Fundamental Mechanisms of Immune Checkpoint Blockade Therapy. Cancer Discov..

[5] Brand M.D. (2010). The sites and topology of mitochondrial superoxide production. Exp. Gerontol..

[6] Bakavayev S., Chetrit N., Zvagelsky T., Mansour R., Vyazmensky M., Barak Z., Israelson A., Engel S. (2019). Cu/Zn-superoxide dismutase and wild-type like fALS SOD1 mutants produce cytotoxic quantities of H_2_O_2_ via cysteine-dependent redox short-circuit. Sci. Rep..

[7] Yan L.J., Levine R.L., Sohal R.S. (1997). Oxidative damage during aging targets mitochondrial aconitase. Proc. Natl. Acad. Sci. USA.

[8] Li Y., Han S., Zhao Y., Yan J., Luo K., Li F., He B., Sun Y., Li F., Liang Y. (2024). A Redox-Triggered Polymeric Nanoparticle for Disrupting Redox Homeostasis and Enhanced Ferroptosis. Small.

[9] Newcomb M., Chandrasena R.E. (2005). Highly reactive electrophilic oxidants in cytochrome P450 catalysis. Biochem. Biophys. Res. Commun..

[10] Qi X., Miao L., Cai Y., Gong L., Ren J. (2013). ROS generated by CYP450, especially CYP2E1, mediate mitochondrial dysfunction induced by tetrandrine in rat hepatocytes. Acta Pharmacol. Sin..

[11] Magnani F., Mattevi A. (2019). Structure and mechanisms of ROS generation by NADPH oxidases. Curr. Opin. Struct. Biol..

[12] Gutteridge J.M., Halliwell B. (1992). Comments on review of Free Radicals in Biology and Medicine, second edition, by Barry Halliwell and John M.C. Gutteridge. Free Radic. Biol. Med..

[13] Roe N.D., Ren J. (2012). Nitric oxide synthase uncoupling: A therapeutic target in cardiovascular diseases. Vascul. Pharmacol..

[14] Liu J., Han X., Zhang T., Tian K., Li Z., Luo F. (2023). Reactive oxygen species (ROS) scavenging biomaterials for anti-inflammatory diseases: From mechanism to therapy. J. Hematol. Oncol..

[15] Basak D., Uddin M.N., Hancock J. (2020). The Role of Oxidative Stress and Its Counteractive Utility in Colorectal Cancer (CRC). Cancers.

[16] de Jager T.L., Cockrell A.E., Du Plessis S.S. (2017). Ultraviolet Light Induced Generation of Reactive Oxygen Species. Adv. Exp. Med. Biol..

[17] Yamamori T., Yasui H., Yamazumi M., Wada Y., Nakamura Y., Nakamura H., Inanami O. (2012). Ionizing radiation induces mitochondrial reactive oxygen species production accompanied by upregulation of mitochondrial electron transport chain function and mitochondrial content under control of the cell cycle checkpoint. Free Radic. Biol. Med..

[18] Mansoor S., Ali A., Kour N., Bornhorst J., AlHarbi K., Rinklebe J., Abd E.M.D., Ahmad P., Chung Y.S. (2023). Heavy Metal Induced Oxidative Stress Mitigation and ROS Scavenging in Plants. Plants.

[19] Zheng F., Goncalves F.M., Abiko Y., Li H., Kumagai Y., Aschner M. (2020). Redox toxicology of environmental chemicals causing oxidative stress. Redox Biol..

[20] Acevedo-Leon D., Monzo-Beltran L., Perez-Sanchez L., Naranjo-Morillo E., Gomez-Abril S.A., Estan-Capell N., Banuls C., Saez G. (2022). Oxidative Stress and DNA Damage Markers in Colorectal Cancer. Int. J. Mol. Sci..

[21] Srinivas U.S., Tan B.W.Q., Vellayappan B.A., Jeyasekharan A.D. (2019). ROS and the DNA damage response in cancer. Redox Biol..

[22] Geng A., Sun J., Tang H., Yu Y., Wang X., Zhang J., Wang X., Sun X., Zhou X., Gao N. (2024). SIRT2 promotes base excision repair by transcriptionally activating OGG1 in an ATM/ATR-dependent manner. Nucleic Acids Res..

[23] Ren M., Gut F., Fan Y., Ma J., Shan X., Yikilmazsoy A., Likhodeeva M., Hopfner K., Zhou C. (2024). Structural basis for human OGG1 processing 8-oxodGuo within nucleosome core particles. Nat. Commun..

[24] Parker A.R., O’Meally R.N., Oliver D.H., Hua L., Nelson W.G., DeWeese T.L., Eshleman J.R. (2002). 8-Hydroxyguanosine repair is defective in some microsatellite stable colorectal cancer cells. Cancer Res..

[25] Casacuberta-Serra S., Gonzalez-Larreategui I., Capitan-Leo D., Soucek L. (2024). MYC and KRAS cooperation: From historical challenges to therapeutic opportunities in cancer. Signal Transduct. Target. Ther..

[26] Ferino A., Rapozzi V., Xodo L.E. (2020). The ROS-KRAS-Nrf2 axis in the control of the redox homeostasis and the intersection with survival-apoptosis pathways: Implications for photodynamic therapy. J. Photochem. Photobiol. B.

[27] Rharass T., Lemcke H., Lantow M., Kuznetsov S.A., Weiss D.G., Panakova D. (2014). Ca^2+^-mediated mitochondrial reactive oxygen species metabolism augments Wnt/beta-catenin pathway activation to facilitate cell differentiation. J. Biol. Chem..

[28] Park M.W., Cha H.W., Kim J., Kim J.H., Yang H., Yoon S., Boonpraman N., Yi S.S., Yoo I.D., Moon J.S. (2021). NOX4 promotes ferroptosis of astrocytes by oxidative stress-induced lipid peroxidation via the impairment of mitochondrial metabolism in Alzheimer’s diseases. Redox Biol..

[29] Monroe T.B., Hertzel A.V., Dickey D.M., Hagen T., Santibanez S.V., Berdaweel I.A., Halley C., Puchalska P., Anderson E.J., Camell C.D. (2024). Lipid peroxidation products induce carbonyl stress, mitochondrial dysfunction, and cellular senescence in human and murine cells. Aging Cell.

[30] Paradies G., Petrosillo G., Pistolese M., Di Venosa N., Federici A., Ruggiero F.M. (2004). Decrease in mitochondrial complex I activity in ischemic/reperfused rat heart: Involvement of reactive oxygen species and cardiolipin. Circ. Res..

[31] Liu T., Sun L., Zhang Y., Wang Y., Zheng J. (2022). Imbalanced GSH/ROS and sequential cell death. J. Biochem. Mol. Toxicol..

[32] Bowie A.G., Moynagh P.N., O’Neill L.A. (1997). Lipid peroxidation is involved in the activation of NF-kappaB by tumor necrosis factor but not interleukin-1 in the human endothelial cell line ECV304. Lack of involvement of H_2_O_2_ in NF-kappaB activation by either cytokine in both primary and transformed endothelial cells. J. Biol. Chem..

[33] Li Y., Zhao T., Li J., Xia M., Li Y., Wang X., Liu C., Zheng T., Chen R., Kan D. (2022). Oxidative Stress and 4-hydroxy-2-nonenal (4-HNE): Implications in the Pathogenesis and Treatment of Aging-related Diseases. J. Immunol. Res..

[34] Jin H., Wang J., Wang Z., Xi M., Xia B., Deng K., Yang J. (2023). Lipid metabolic reprogramming in tumor microenvironment: From mechanisms to therapeutics. J. Hematol. Oncol..

[35] Houglum K., Filip M., Witztum J.L., Chojkier M. (1990). Malondialdehyde and 4-hydroxynonenal protein adducts in plasma and liver of rats with iron overload. J. Clin. Investig..

[36] Yao M., Kitamura F., Han Y., Du H., Julian M.D., Xiao H. (2022). Adverse effects of linoleic acid: Influence of lipid oxidation on lymphatic transport of citrus flavonoid and enterocyte morphology. Food Chem..

[37] Butterfield D.A., Gu L., Di Domenico F., Robinson R.A. (2014). Mass spectrometry and redox proteomics: Applications in disease. Mass. Spectrom. Rev..

[38] Cumming R.C., Andon N.L., Haynes P.A., Park M., Fischer W.H., Schubert D. (2004). Protein disulfide bond formation in the cytoplasm during oxidative stress. J. Biol. Chem..

[39] Isoda T., Nakatsu Y., Yamauchi K., Piao J., Yao T., Honda H., Nakabeppu Y., Tsuzuki T. (2014). Abnormality in Wnt signaling is causatively associated with oxidative stress-induced intestinal tumorigenesis in MUTYH-null mice. Int. J. Biol. Sci..

[40] Iqbal M.J., Kabeer A., Abbas Z., Siddiqui H.A., Calina D., Sharifi-Rad J., Cho W.C. (2024). Interplay of oxidative stress, cellular communication and signaling pathways in cancer. Cell Commun. Signal.

[41] Shi T., Dansen T.B. (2020). Reactive Oxygen Species Induced p53 Activation: DNA Damage, Redox Signaling, or Both?. Antioxid Redox Signal.

[42] Vilchis-Landeros M.M., Vazquez-Meza H., Vazquez-Carrada M., Uribe-Ramirez D., Matuz-Mares D. (2024). Antioxidant Enzymes and Their Potential Use in Breast Cancer Treatment. Int. J. Mol. Sci..

[43] Chao W.W., Su C.C., Peng H.Y., Chou S.T. (2017). Melaleuca quinquenervia essential oil inhibits alpha-melanocyte-stimulating hormone-induced melanin production and oxidative stress in B16 melanoma cells. Phytomedicine.

[44] Kwiterovich P.O.J. (2000). The metabolic pathways of high-density lipoprotein, low-density lipoprotein, and triglycerides: A current review. Am. J. Cardiol..

[45] Sottero B., Gargiulo S., Russo I., Barale C., Poli G., Cavalot F. (2015). Postprandial Dysmetabolism and Oxidative Stress in Type 2 Diabetes: Pathogenetic Mechanisms and Therapeutic Strategies. Med. Res. Rev..

[46] Ma C., Xie J., Luo C., Yin H., Li R., Wang X., Xiong W., Zhang T., Jiang P., Qi W. (2019). OxLDL promotes lymphangiogenesis and lymphatic metastasis in gastric cancer by upregulating VEGF-C expression and secretion. Int. J. Oncol..

[47] Suzuki K., Ito Y., Wakai K., Kawado M., Hashimoto S., Toyoshima H., Kojima M., Tokudome S., Hayakawa N., Watanabe Y. (2004). Serum oxidized low-density lipoprotein levels and risk of colorectal cancer: A case-control study nested in the Japan Collaborative Cohort Study. Cancer Epidemiol. Biomark. Prev..

[48] Delimaris I., Faviou E., Antonakos G., Stathopoulou E., Zachari A., Dionyssiou-Asteriou A. (2007). Oxidized LDL, serum oxidizability and serum lipid levels in patients with breast or ovarian cancer. Clin. Biochem..

[49] Balzan S., Lubrano V. (2018). LOX-1 receptor: A potential link in atherosclerosis and cancer. Life Sci..

[50] Moghadam S.G., Ebrahimpour M., Alavizadeh S.H., Kesharwani P., Sahebkar A. (2024). The association between oxidized low-density lipoprotein and cancer: An emerging targeted therapeutic approach?. Bioorg Med. Chem. Lett..

[51] Heermeier K., Schneider R., Heinloth A., Wanner C., Dimmeler S., Galle J. (1999). Oxidative stress mediates apoptosis induced by oxidized low-density lipoprotein and oxidized lipoprotein(a). Kidney Int..

[52] Holvoet P., Lee D.H., Steffes M., Gross M., Jacobs D.J. (2008). Association between circulating oxidized low-density lipoprotein and incidence of the metabolic syndrome. JAMA.

[53] Li D., Liu L., Chen H., Sawamura T., Ranganathan S., Mehta J.L. (2003). LOX-1 mediates oxidized low-density lipoprotein-induced expression of matrix metalloproteinases in human coronary artery endothelial cells. Circulation.

[54] Murdocca M., Mango R., Pucci S., Biocca S., Testa B., Capuano R., Paolesse R., Sanchez M., Orlandi A., di Natale C. (2016). The lectin-like oxidized LDL receptor-1: A new potential molecular target in colorectal cancer. Oncotarget.

[55] Keshk W.A., Zineldeen D.H., Wasfy R.E.L., El-Khadrawy O.H. (2014). Fatty acid synthase/oxidized low-density lipoprotein as metabolic oncogenes linking obesity to colon cancer via NF-kappa B in Egyptians. Med. Oncol..

[56] Zheng S., Chen H., Sha W., Chen X., Yin J., Zhu X., Zheng Z., Ma J. (2022). Oxidized low-density lipoprotein stimulates CD206 positive macrophages upregulating CD44 and CD133 expression in colorectal cancer with high-fat diet. World J. Gastroenterol..

[57] Aoki Y., Dai H., Furuta F., Akamatsu T., Oshima T., Takahashi N., Goto Y.I., Oka A., Itoh M. (2023). LOX-1 mediates inflammatory activation of microglial cells through the p38-MAPK/NF-kappaB pathways under hypoxic-ischemic conditions. Cell Commun. Signal.

[58] Chen G., Zhou Y., Zhang W., Qin Y., Wei B., Sun Y., Chen Y. (2021). Methyl-beta-cyclodextrin suppresses the monocyte-endothelial adhesion triggered by lipopolysaccharide (LPS) or oxidized low-density lipoprotein (oxLDL). Pharm. Biol..

[59] Carty F., Layzell S., Barbarulo A., Islam F., Webb L.V., Seddon B. (2023). IKK promotes naive T cell survival by repressing RIPK1-dependent apoptosis and activating NF-kappaB. Sci. Signal.

[60] Cao Y., Yi Y., Han C., Shi B. (2024). NF-kappaB signaling pathway in tumor microenvironment. Front. Immunol..

[61] AboZaid O., Abdel-Maksoud M.A., Saleh I.A., El-Tayeb M.A., El-Sonbaty S.M., Shoker F.E., Salem M.A., Emad A.M., Mani S., Deva M.R.A. (2024). Targeting the NF-kappaB p65/Bcl-2 signaling pathway in hepatic cellular carcinoma using radiation assisted synthesis of zinc nanoparticles coated with naturally isolated gallic acid. Biomed. Pharmacother..

[62] Aguilera T.A., Giaccia A.J. (2017). Molecular Pathways: Oncologic Pathways and Their Role in T-cell Exclusion and Immune Evasion-A New Role for the AXL Receptor Tyrosine Kinase. Clin. Cancer Res..

[63] Hagemann T., Biswas S.K., Lawrence T., Sica A., Lewis C.E. (2009). Regulation of macrophage function in tumors: The multifaceted role of NF-kappaB. Blood.

[64] Nakai K., Lin H., Yamano S., Tanaka S., Kitamoto S., Saitoh H., Sakuma K., Kurauchi J., Akter E., Konno M. (2023). Wnt activation disturbs cell competition and causes diffuse invasion of transformed cells through NF-kappaB-MMP21 pathway. Nat. Commun..

[65] Bahrami A., Khalaji A., Bahri Najafi M., Sadati S., Raisi A., Abolhassani A., Eshraghi R., Khaksary Mahabady M., Rahimian N., Mirzaei H. (2024). NF-kappaB pathway and angiogenesis: Insights into colorectal cancer development and therapeutic targets. Eur. J. Med. Res..

[66] Khaidakov M., Mehta J.L. (2012). Oxidized LDL triggers pro-oncogenic signaling in human breast mammary epithelial cells partly via stimulation of MiR-21. PLoS ONE.

[67] Khezri M.R., Jafari R., Yousefi K., Zolbanin N.M. (2022). The PI3K/AKT signaling pathway in cancer: Molecular mechanisms and possible therapeutic interventions. Exp. Mol. Pathol..

[68] Rahmani M., Nkwocha J., Hawkins E., Pei X., Parker R.E., Kmieciak M., Leverson J.D., Sampath D., Ferreira-Gonzalez A., Grant S. (2018). Cotargeting BCL-2 and PI3K Induces BAX-Dependent Mitochondrial Apoptosis in AML Cells. Cancer Res..

[69] Hassan D., Menges C.W., Testa J.R., Bellacosa A. (2024). AKT kinases as therapeutic targets. J. Exp. Clin. Cancer Res..

[70] Mao D., Zhou Z., Chen H., Liu X., Li D., Chen X., He Y., Liu M., Zhang C. (2023). Pleckstrin-2 promotes tumour immune escape from NK cells by activating the MT1-MMP-MICA signalling axis in gastric cancer. Cancer Lett..

[71] Zhang H., Schaefer A., Wang Y., Hodge R.G., Blake D.R., Diehl J.N., Papageorge A.G., Stachler M.D., Liao J., Zhou J. (2020). Gain-of-Function RHOA Mutations Promote Focal Adhesion Kinase Activation and Dependency in Diffuse Gastric Cancer. Cancer Discov..

[72] Zhang W., Liu H.T. (2002). MAPK signal pathways in the regulation of cell proliferation in mammalian cells. Cell Res..

[73] Wang H.H., Hsieh H.L., Wu C.Y., Sun C.C., Yang C.M. (2009). Oxidized low-density lipoprotein induces matrix metalloproteinase-9 expression via a p42/p44 and JNK-dependent AP-1 pathway in brain astrocytes. Glia.

[74] Zhang K., Meng X., Kong J., Liu F.F., Yang J.M., Gao F., Zhang Y., Zhang C. (2013). Simvastatin increases Prolyl-4-Hydroxylase alpha1 expression in atherosclerotic plaque and oxLDL-stimulated human aortic smooth muscle cells via p38 MAPK and ERK1/2 signaling. J. Mol. Cell Cardiol..

[75] Lucas R.M., Luo L., Stow J.L. (2022). ERK1/2 in immune signalling. Biochem. Soc. Trans..

[76] Damianova R., Stefanova N., Cukierman E., Momchilova A., Pankov R. (2008). Three-dimensional matrix induces sustained activation of ERK1/2 via Src/Ras/Raf signaling pathway. Cell Biol. Int..

[77] Aksamitiene E., Achanta S., Kolch W., Kholodenko B.N., Hoek J.B., Kiyatkin A. (2011). Prolactin-stimulated activation of ERK1/2 mitogen-activated protein kinases is controlled by PI3-kinase/Rac/PAK signaling pathway in breast cancer cells. Cell Signal.

[78] Pan C.C., Bloodworth J.C., Mythreye K., Lee N.Y. (2012). Endoglin inhibits ERK-induced c-Myc and cyclin D1 expression to impede endothelial cell proliferation. Biochem. Biophys. Res. Commun..

[79] Tabei Y., Nakajima Y. (2024). IL-1beta-activated PI3K/AKT and MEK/ERK pathways coordinately promote induction of partial epithelial-mesenchymal transition. Cell Commun. Signal.

[80] Grimes K.M., Maillet M., Swoboda C.O., Bowers S.L.K., Millay D.P., Molkentin J.D. (2024). MEK1-ERK1/2 signaling regulates the cardiomyocyte non-sarcomeric actin cytoskeletal network. Am. J. Physiol. Heart Circ. Physiol..

[81] Martinez-Limon A., Joaquin M., Caballero M., Posas F., de Nadal E. (2020). The p38 Pathway: From Biology to Cancer Therapy. Int. J. Mol. Sci..

[82] Zhu L., Lama S., Tu L., Dusting G.J., Wang J.H., Liu G.S. (2021). TAK1 signaling is a potential therapeutic target for pathological angiogenesis. Angiogenesis.

[83] Uhlig U., Haitsma J.J., Goldmann T., Poelma D.L., Lachmann B., Uhlig S. (2002). Ventilation-induced activation of the mitogen-activated protein kinase pathway. Eur. Respir. J..

[84] Ronkina N., Menon M.B., Schwermann J., Arthur J.S., Legault H., Telliez J.B., Kayyali U.S., Nebreda A.R., Kotlyarov A., Gaestel M. (2011). Stress induced gene expression: A direct role for MAPKAP kinases in transcriptional activation of immediate early genes. Nucleic Acids Res..

[85] Rajashekhar G., Kamocka M., Marin A., Suckow M.A., Wolter W.R., Badve S., Sanjeevaiah A.R., Pumiglia K., Rosen E., Clauss M. (2011). Pro-inflammatory angiogenesis is mediated by p38 MAP kinase. J. Cell Physiol..

[86] De Zutter G.S., Davis R.J. (2001). Pro-apoptotic gene expression mediated by the p38 mitogen-activated protein kinase signal transduction pathway. Proc. Natl. Acad. Sci. USA.

[87] Ma Y., Dong X., Wang Y., Wang Z., Xie Y., Zhang W., Pan D., Zhou H., Xu B. (2024). New findings on post-mortem chicken quality changes: The ROS-influenced MAPK-JNK signaling pathway affects chicken quality by regulating muscle cell apoptosis. Food Chem..

[88] Pu Z.Q., Yu T.F., Liu D., Jin C.W., Sadiq E., Qiao X., Li X., Chen Y., Zhang J., Tian M. (2021). NR4A1 enhances MKP7 expression to diminish JNK activation induced by ROS or ER-stress in pancreatic beta cells for surviving. Cell Death Discov..

[89] Morton S., Davis R.J., McLaren A., Cohen P. (2003). A reinvestigation of the multisite phosphorylation of the transcription factor c-Jun. EMBO J..

[90] Renoux F., Stellato M., Haftmann C., Vogetseder A., Huang R., Subramaniam A., Becker M.O., Blyszczuk P., Becher B., Distler J. (2020). The AP1 Transcription Factor Fosl2 Promotes Systemic Autoimmunity and Inflammation by Repressing Treg Development. Cell Rep..

[91] Sahu S.K., Garding A., Tiwari N., Thakurela S., Toedling J., Gebhard S., Ortega F., Schmarowski N., Berninger B., Nitsch R. (2015). JNK-dependent gene regulatory circuitry governs mesenchymal fate. EMBO J..

[92] Hicklin D.J., Ellis L.M. (2005). Role of the vascular endothelial growth factor pathway in tumor growth and angiogenesis. J. Clin. Oncol..

[93] Cao M., Wang Y., Lu G., Qi H., Li P., Dai X., Lu J. (2022). Classical Angiogenic Signaling Pathways and Novel Anti-Angiogenic Strategies for Colorectal Cancer. Curr. Issues Mol. Biol..

[94] Kiriakidis S., Andreakos E., Monaco C., Foxwell B., Feldmann M., Paleolog E. (2003). VEGF expression in human macrophages is NF-kappaB-dependent: Studies using adenoviruses expressing the endogenous NF-kappaB inhibitor IkappaBalpha and a kinase-defective form of the IkappaB kinase 2. J. Cell Sci..

[95] Wang L., Zhao L., Zhang L., Jing X., Zhang Y., Shao S., Zhao X., Luo M. (2021). Vascular endothelial growth factor promotes cancer stemness of triple-negative breast cancer via MAPK/ERK pathway. J. South. Med. Univ..

[96] Ma C., Huang W., Wang H., Yao W., Liang M., Yu G., Zhou X. (2022). Oxidized LDL promotes EMS-induced angiogenesis by increasing VEGF-A expression and secretion by endometrial cells. Mol. Med..

[97] Cohen T., Nahari D., Cerem L.W., Neufeld G., Levi B.Z. (1996). Interleukin 6 induces the expression of vascular endothelial growth factor. J. Biol. Chem..

[98] Yin Y., Wang S., Sun Y., Matt Y., Colburn N.H., Shu Y., Han X. (2009). JNK/AP-1 pathway is involved in tumor necrosis factor-alpha induced expression of vascular endothelial growth factor in MCF7 cells. Biomed. Pharmacother..

[99] Le Guelte A., Dwyer J., Gavard J. (2011). Jumping the barrier: VE-cadherin, VEGF and other angiogenic modifiers in cancer. Biol. Cell.

[100] Yang Y., Cao Y. (2022). The impact of VEGF on cancer metastasis and systemic disease. Semin. Cancer Biol..

[101] Horikawa N., Abiko K., Matsumura N., Hamanishi J., Baba T., Yamaguchi K., Yoshioka Y., Koshiyama M., Konishi I. (2017). Expression of Vascular Endothelial Growth Factor in Ovarian Cancer Inhibits Tumor Immunity through the Accumulation of Myeloid-Derived Suppressor Cells. Clin. Cancer Res..

[102] Gabrilovich D.I., Chen H.L., Girgis K.R., Cunningham H.T., Meny G.M., Nadaf S., Kavanaugh D., Carbone D.P. (1996). Production of vascular endothelial growth factor by human tumors inhibits the functional maturation of dendritic cells. Nat. Med..

[103] Suzuki H., Onishi H., Wada J., Yamasaki A., Tanaka H., Nakano K., Morisaki T., Katano M. (2010). VEGFR2 is selectively expressed by FOXP3high CD4+ Treg. Eur. J. Immunol..

[104] Voron T., Colussi O., Marcheteau E., Pernot S., Nizard M., Pointet A.L., Latreche S., Bergaya S., Benhamouda N., Tanchot C. (2015). VEGF-A modulates expression of inhibitory checkpoints on CD8+ T cells in tumors. J. Exp. Med..

[105] Hordijk P.L. (2006). Regulation of NADPH oxidases: The role of Rac proteins. Circ. Res..

[106] Cho S.Y., Kim J.S., Eun H.S., Kang S.H., Lee E.S., Kim S.H., Sung J.K., Lee B.S., Jeong H.Y., Moon H.S. (2018). Expression of NOX Family Genes and Their Clinical Significance in Colorectal Cancer. Dig. Dis. Sci..

[107] Guo H.Z., Feng R.X., Zhang Y.J., Yu Y.H., Lu W., Liu J.J., Yang S.X., Zhao C., Zhang Z.L., Yu S.H. (2024). A CD36-dependent non-canonical lipid metabolism program promotes immune escape and resistance to hypomethylating agent therapy in AML. Cell Rep. Med..

[108] Bai Z., Zhou Y., Ye Z., Xiong J., Lan H., Wang F. (2021). Tumor-Infiltrating Lymphocytes in Colorectal Cancer: The Fundamental Indication and Application on Immunotherapy. Front. Immunol..

[109] Leowattana W., Leowattana P., Leowattana T. (2023). Systemic treatment for metastatic colorectal cancer. World J. Gastroenterol..

[110] Guven D.C., Kavgaci G., Erul E., Syed M.P., Magge T., Saeed A., Yalcin S., Sahin I.H. (2024). The Efficacy of Immune Checkpoint Inhibitors in Microsatellite Stable Colorectal Cancer: A Systematic Review. Oncologist.

[111] Wu H., Deng M., Xue D., Guo R., Zhang C., Gao J., Li H. (2024). PD-1/PD-L1 inhibitors for early and middle stage microsatellite high-instability and stable colorectal cancer: A review. Int. J. Color. Dis..

[112] Lizardo D.Y., Kuang C., Hao S., Yu J., Huang Y., Zhang L. (2020). Immunotherapy efficacy on mismatch repair-deficient colorectal cancer: From bench to bedside. Biochim. Biophys. Acta Rev. Cancer.

[113] Ding K., Mou P., Wang Z., Liu S., Liu J., Lu H., Yu G. (2023). The next bastion to be conquered in immunotherapy: Microsatellite stable colorectal cancer. Front. Immunol..

[114] Zhu Y., Liu J. (2021). The Role of Neoantigens in Cancer Immunotherapy. Front. Oncol..

[115] Andarawi S., Vodickova L., Uttarilli A., Hanak P., Vodicka P. (2025). Defective DNA repair: A putative nexus linking immunological diseases, neurodegenerative disorders, and cancer. Mutagenesis.

[116] Poloni C., Schonhofer C., Ivison S., Levings M.K., Steiner T.S., Cook L. (2023). T-cell activation-induced marker assays in health and disease. Immunol. Cell Biol..

[117] Zhou Y., Zhou Y., Liu S., Wang J., Ji R., Yan X. (2021). Prognostic and immunomodulatory effects of PIM1 in colorectal carcinoma. J. Buon.

[118] Westcott P.M.K., Sacks N.J., Schenkel J.M., Ely Z.A., Smith O., Hauck H., Jaeger A.M., Zhang D., Backlund C.M., Beytagh M.C. (2021). Low neoantigen expression and poor T-cell priming underlie early immune escape in colorectal cancer. Nat. Cancer.

[119] Ghiringhelli F., Fumet J. (2019). Is There a Place for Immunotherapy for Metastatic Microsatellite Stable Colorectal Cancer?. Front. Immunol..

[120] Chen E., Zhou W. (2025). Immunotherapy in Microsatellite-Stable Colorectal Cancer: Strategies to Overcome Resistance. Crit. Rev. Oncol. Hematol..

[121] Lu Y., Robbins P.F. (2016). Targeting neoantigens for cancer immunotherapy. Int. Immunol..

[122] Jwo S., Ng S., Li C., Chen S., Chen L., Liu P., Wang H., Lin J., Ko C., Lee C. (2025). Dual prophylactic and therapeutic potential of iPSC-based vaccines and neoantigen discovery in colorectal cancer. Theranostics.

[123] Marabelle A., Fakih M., Lopez J., Shah M., Shapira-Frommer R., Nakagawa K., Chung H.C., Kindler H.L., Lopez-Martin J.A., Miller W.H.J. (2020). Association of tumour mutational burden with outcomes in patients with advanced solid tumours treated with pembrolizumab: Prospective biomarker analysis of the multicohort, open-label, phase 2 KEYNOTE-158 study. Lancet Oncol..

[124] Xu D., Geng J., Gao Z., Fan C., Zhang B., Han X., He L., Dai L., Gao S., Yang Z. (2025). To explore the potential combined treatment strategy for colorectal cancer: Inhibition of cancer stem cells and enhancement of intestinal immune microenvironment. Eur. J. Pharmacol..

[125] Toor S.M., Sasidharan Nair V., Murshed K., Abu Nada M., Elkord E. (2021). Tumor-Infiltrating Lymphoid Cells in Colorectal Cancer Patients with Varying Disease Stages and Microsatellite Instability-High/Stable Tumors. Vaccines.

[126] Chen L., Jiang X., Li Y., Zhang Q., Li Q., Zhang X., Zhang M., Yu Q., Gao D. (2022). How to overcome tumor resistance to anti-PD-1/PD-L1 therapy by immunotherapy modifying the tumor microenvironment in MSS CRC. Clin Immunol..

[127] Bai J., Chen H., Bai X. (2021). Relationship between microsatellite status and immune microenvironment of colorectal cancer and its application to diagnosis and treatment. J. Clin. Lab. Anal..

[128] Mlecnik B., Tosolini M., Charoentong P., Kirilovsky A., Bindea G., Berger A., Camus M., Gillard M., Bruneval P., Fridman W. (2010). Biomolecular network reconstruction identifies T-cell homing factors associated with survival in colorectal cancer. Gastroenterology.

[129] Zhang L., Yu X., Zheng L., Zhang Y., Li Y., Fang Q., Gao R., Kang B., Zhang Q., Huang J.Y. (2018). Lineage tracking reveals dynamic relationships of T cells in colorectal cancer. Nature.

[130] Chow M.T., Ozga A.J., Servis R.L., Frederick D.T., Lo J.A., Fisher D.E., Freeman G.J., Boland G.M., Luster A.D. (2019). Intratumoral Activity of the CXCR3 Chemokine System Is Required for the Efficacy of Anti-PD-1 Therapy. Immunity.

[131] Gorria T., Sierra-Boada M., Rojas M., Figueras C., Marin S., Madurga S., Cascante M., Maurel J. (2025). Metabolic Singularities in Microsatellite-Stable Colorectal Cancer: Identifying Key Players in Immunosuppression to Improve the Immunotherapy Response. Cancers.

[132] Mu X., Shi W., Xu Y., Xu C., Zhao T., Geng B., Yang J., Pan J., Hu S., Zhang C. (2018). Tumor-derived lactate induces M2 macrophage polarization via the activation of the ERK/STAT3 signaling pathway in breast cancer. Cell Cycle.

[133] Cao J., Liu C. (2024). Mechanistic studies of tumor-associated macrophage immunotherapy. Front. Immunol..

[134] Cheng Y., Han X., Lai X., Wei X. (2024). Liposomal honokiol inhibits non-small cell lung cancer progression and enhances PD-1 blockade via suppressing M2 macrophages polarization. Phytomedicine.

[135] Cai J., Song L., Zhang F., Wu S., Zhu G., Zhang P., Chen S., Du J., Wang B., Cai Y. (2024). Targeting SRSF10 might inhibit M2 macrophage polarization and potentiate anti-PD-1 therapy in hepatocellular carcinoma. Cancer Commun..

[136] Sezginer O., Unver N. (2024). Dissection of pro-tumoral macrophage subtypes and immunosuppressive cells participating in M2 polarization. Inflamm. Res..

[137] Gu Y., Mi Y., Cao Y., Yu K., Zhang Z., Lian P., Li D., Qin J., Zhao S. (2025). The lncRNA MIR181A1HG in extracellular vesicles derived from highly metastatic colorectal cancer cells promotes liver metastasis by remodeling the extracellular matrix and recruiting myeloid-derived suppressor cells. Cell Biosci..

[138] Ding X., Zhang L., Fan M., Li L. (2024). TME-NET: An interpretable deep neural network for predicting pan-cancer immune checkpoint inhibitor responses. Brief. Bioinform..

[139] Mu Q., Najafi M. (2021). Modulation of the tumor microenvironment (TME) by melatonin. Eur. J. Pharmacol..

[140] Ma W., Li Z., Wu Z., Liu F., Wang J., Shi Y., Jin Y., Li F. (2023). PI3K-CCL2-CCR2-MDSCs axis: A potential pathway for tumor Clostridia-promoted CD 8(+) T lymphocyte infiltration in bile tract cancers. Neoplasia.

[141] Ding D., Zhong H., Liang R., Lan T., Zhu X., Huang S., Wang Y., Shao J., Shuai X., Wei B. (2021). Multifunctional Nanodrug Mediates Synergistic Photodynamic Therapy and MDSCs-Targeting Immunotherapy of Colon Cancer. Adv. Sci..

[142] OuYang L., Wu X., Ye S., Zhang R., Li Z., Liao W., Pan Z., Zheng L., Zhang X., Wang Z. (2015). Tumor-induced myeloid-derived suppressor cells promote tumor progression through oxidative metabolism in human colorectal cancer. J. Transl. Med..

[143] Zhou L., Jiang Y., Li X., Zhang J., Li S., Wei L., Zhang H., Zhou G., Chen X., Sun L. (2024). Myeloid-derived suppressor cells-induced exhaustion of CD8 + T-cell participates in rejection after liver transplantation. Cell Death Dis..

[144] Chen Y., Liang Z., Lai M. (2024). Targeting the devil: Strategies against cancer-associated fibroblasts in colorectal cancer. Transl. Res..

[145] Chung J.Y., Chan M.K., Li J.S., Chan A.S., Tang P.C., Leung K., To K., Lan H., Tang P.M. (2021). TGF-beta Signaling: From Tissue Fibrosis to Tumor Microenvironment. Int. J. Mol. Sci..

[146] Yin J., Zhu W., Feng S., Yan P., Qin S. (2024). The role of cancer-associated fibroblasts in the invasion and metastasis of colorectal cancer. Front. Cell Dev. Biol..

[147] Calon A., Tauriello D.V.F., Batlle E. (2014). TGF-beta in CAF-mediated tumor growth and metastasis. Semin. Cancer Biol..

[148] Amer H.T., Stein U., El Tayebi H.M. (2022). The Monocyte, a Maestro in the Tumor Microenvironment (TME) of Breast Cancer. Cancers.

[149] Deryugina E.I., Zajac E., Juncker-Jensen A., Kupriyanova T.A., Welter L., Quigley J.P. (2014). Tissue-infiltrating neutrophils constitute the major in vivo source of angiogenesis-inducing MMP-9 in the tumor microenvironment. Neoplasia.

[150] Masucci M.T., Minopoli M., Del Vecchio S., Carriero M.V. (2020). The Emerging Role of Neutrophil Extracellular Traps (NETs) in Tumor Progression and Metastasis. Front. Immunol..

[151] Mahaki H., Nobari S., Tanzadehpanah H., Babaeizad A., Kazemzadeh G., Mehrabzadeh M., Valipour A., Yazdinezhad N., Manoochehri H., Yang P. (2025). Targeting VEGF signaling for tumor microenvironment remodeling and metastasis inhibition: Therapeutic strategies and insights. Biomed. Pharmacother..

[152] Legitimo A., Consolini R., Failli A., Orsini G., Spisni R. (2014). Dendritic cell defects in the colorectal cancer. Hum. Vaccin. Immunother..

[153] Gao M., Zhang X., Li D., He P., Tian W., Zeng B. (2016). Expression analysis and clinical significance of eIF4E, VEGF-C, E-cadherin and MMP-2 in colorectal adenocarcinoma. Oncotarget.

[154] Kim C.G., Jang M., Kim Y., Leem G., Kim K.H., Lee H., Kim T., Choi S.J., Kim H., Han J.W. (2019). VEGF-A drives TOX-dependent T cell exhaustion in anti-PD-1-resistant microsatellite stable colorectal cancers. Sci. Immunol..

[155] Sun X., Zhan L., Chen Y., Wang G., He L., Wang Q., Zhou F., Yang F., Wu J., Wu Y. (2018). Increased mtDNA copy number promotes cancer progression by enhancing mitochondrial oxidative phosphorylation in microsatellite-stable colorectal cancer. Signal Transduct. Target. Ther..

[156] Chen M., Liu H., Liang W., Huang P., Ye F., Cai Y., Liang Z., Xiong L., Kang L., Huang L. (2024). Mitochondrial DNA copy number plays opposing roles in T-lymphocyte infiltration of colorectal cancer based on mismatch repair status: New directions for immunotherapy?. Br. J. Cancer.

[157] Wenes M., Jaccard A., Wyss T., Maldonado-Perez N., Teoh S.T., Lepez A., Renaud F., Franco F., Waridel P., Yacoub Maroun C. (2022). The mitochondrial pyruvate carrier regulates memory T cell differentiation and antitumor function. Cell Metab..

[158] Guo J., Zheng L., Liu W., Wang X., Wang Z., Wang Z., French A.J., Kang D., Chen L., Thibodeau S.N. (2011). Frequent truncating mutation of TFAM induces mitochondrial DNA depletion and apoptotic resistance in microsatellite-unstable colorectal cancer. Cancer Res..

[159] Tong H., Zhang L., Gao J., Wen S., Zhou H., Feng S. (2017). Methylation of mitochondrial DNA displacement loop region regulates mitochondrial copy number in colorectal cancer. Mol. Med. Rep..

[160] Noman M.Z., Desantis G., Janji B., Hasmim M., Karray S., Dessen P., Bronte V., Chouaib S. (2014). PD-L1 is a novel direct target of HIF-1alpha, and its blockade under hypoxia enhanced MDSC-mediated T cell activation. J. Exp. Med..

[161] Ma S., Deng W., Liu J., Mao L., Yu G., Bu L., Kulkarni A.B., Zhang W., Sun Z. (2017). Blockade of adenosine A2A receptor enhances CD8(+) T cells response and decreases regulatory T cells in head and neck squamous cell carcinoma. Mol. Cancer.

[162] Xia C., Yin S., To K.K.W., Fu L. (2023). CD39/CD73/A2AR pathway and cancer immunotherapy. Mol. Cancer.

[163] Karki S., Umar S., Kasi A. (2020). Treating Colorectal Cancer with Immunotherapy: Implications for Single versus Combination Therapy. Curr. Colorectal Cancer Rep..

[164] Yang X., Wu H. (2024). RAS signaling in carcinogenesis, cancer therapy and resistance mechanisms. J. Hematol. Oncol..

[165] Adachi Y., Shibai Y., Mitsushita J., Shang W.H., Hirose K., Kamata T. (2008). Oncogenic Ras upregulates NADPH oxidase 1 gene expression through MEK-ERK-dependent phosphorylation of GATA-6. Oncogene.

[166] Wang X., Fu Y., Liu X., Feng G., Xiong D., Mu G., Chen F. (2018). ROS Promote OxLDL-Induced Platelet Activation by Up-Regulating Autophagy Through the Inhibition of the PI3K/AKT/mTOR Pathway. Cell Physiol. Biochem..

[167] Jin B., Robertson K.D. (2013). DNA methyltransferases, DNA damage repair, and cancer. Adv. Exp. Med. Biol..

[168] Wang C., Zhou X., Li W., Li M., Tu T., Ba X., Wu Y., Huang Z., Fan G., Zhou G. (2017). Macrophage migration inhibitory factor promotes osteosarcoma growth and lung metastasis through activating the RAS/MAPK pathway. Cancer Lett..

[169] Hasan M.N., Capuk O., Patel S.M., Sun D. (2022). The Role of Metabolic Plasticity of Tumor-Associated Macrophages in Shaping the Tumor Microenvironment Immunity. Cancers.

[170] Yuan A., Hsiao Y., Chen H., Chen H., Ho C., Chen Y., Liu Y., Hong T., Yu S., Chen J.J.W. (2015). Opposite Effects of M1 and M2 Macrophage Subtypes on Lung Cancer Progression. Sci. Rep..

[171] Pearce E.L., Pearce E.J. (2013). Metabolic pathways in immune cell activation and quiescence. Immunity.

[172] Hou Y., Wei D., Zhang Z., Guo H., Li S., Zhang J., Zhang P., Zhang L., Zhao Y. (2022). FABP5 controls macrophage alternative activation and allergic asthma by selectively programming long-chain unsaturated fatty acid metabolism. Cell Rep..

[173] Jin R., Neufeld L., McGaha T.L. (2025). Linking macrophage metabolism to function in the tumor microenvironment. Nat. Cancer.

[174] Su P., Wang Q., Bi E., Ma X., Liu L., Yang M., Qian J., Yi Q. (2020). Enhanced Lipid Accumulation and Metabolism Are Required for the Differentiation and Activation of Tumor-Associated Macrophages. Cancer Res..

[175] Balic J.J., Albargy H., Luu K., Kirby F.J., Jayasekara W.S.N., Mansell F., Garama D.J., De Nardo D., Baschuk N., Louis C. (2020). STAT3 serine phosphorylation is required for TLR4 metabolic reprogramming and IL-1beta expression. Nat. Commun..

[176] Zhang H., Caudle Y., Wheeler C., Zhou Y., Stuart C., Yao B., Yin D. (2018). TGF-beta1/Smad2/3/Foxp3 signaling is required for chronic stress-induced immune suppression. J. Neuroimmunol..

[177] Koppelman B., Neefjes J.J., de Vries J.E., de Waal Malefyt R. (1997). Interleukin-10 down-regulates MHC class II alphabeta peptide complexes at the plasma membrane of monocytes by affecting arrival and recycling. Immunity.

[178] Yi M., Li T., Niu M., Wu Y., Zhao Z., Wu K. (2022). TGF-beta: A novel predictor and target for anti-PD-1/PD-L1 therapy. Front. Immunol..

[179] McKillop I.H., Girardi C.A., Thompson K.J. (2019). Role of fatty acid binding proteins (FABPs) in cancer development and progression. Cell Signal.

[180] Huang B., Lang X., Li X. (2022). The role of IL-6/JAK2/STAT3 signaling pathway in cancers. Front. Oncol..

[181] Wu F., Yang J., Liu J., Wang Y., Mu J., Zeng Q., Deng S., Zhou H. (2021). Signaling pathways in cancer-associated fibroblasts and targeted therapy for cancer. Signal Transduct. Target. Ther..

[182] Li Q., Yang Y., Jiang X., Jin Y., Wu J., Qin Y., Qi X., Cheng Y., Mao Y., Hua D. (2019). The combined expressions of B7H4 and ACOT4 in cancer-associated fibroblasts are related to poor prognosis in patients with gastric carcinoma. Int. J. Clin. Exp. Pathol..

[183] Zhang H., Deng T., Liu R., Ning T., Yang H., Liu D., Zhang Q., Lin D., Ge S., Bai M. (2020). CAF secreted miR-522 suppresses ferroptosis and promotes acquired chemo-resistance in gastric cancer. Mol. Cancer.

[184] Singh N.K., Rao G.N. (2019). Emerging role of 12/15-Lipoxygenase (ALOX15) in human pathologies. Prog. Lipid Res..

[185] Bleve A., Durante B., Sica A., Consonni F.M. (2020). Lipid Metabolism and Cancer Immunotherapy: Immunosuppressive Myeloid Cells at the Crossroad. Int. J. Mol. Sci..

[186] Wang H., Zhou F., Qin W., Yang Y., Li X., Liu R. (2025). Metabolic regulation of myeloid-derived suppressor cells in tumor immune microenvironment: Targets and therapeutic strategies. Theranostics.

[187] Li Q., Xiang M. (2022). Metabolic reprograming of MDSCs within tumor microenvironment and targeting for cancer immunotherapy. Acta Pharmacol. Sin..

[188] Rozpedek W., Pytel D., Mucha B., Leszczynska H., Diehl J.A., Majsterek I. (2016). The Role of the PERK/eIF2alpha/ATF4/CHOP Signaling Pathway in Tumor Progression During Endoplasmic Reticulum Stress. Curr. Mol. Med..

[189] Ziani L., Chouaib S., Thiery J. (2018). Alteration of the Antitumor Immune Response by Cancer-Associated Fibroblasts. Front. Immunol..

[190] Li P., Chen Y., Xiang Y., Guo R., Li X., Liu J., Zhou Y., Fu X. (2024). 17beta-estradiol promotes myeloid-derived suppressor cells functions and alleviates inflammatory bowel disease by activation of Stat3 and NF-kappaB signalings. J. Steroid Biochem. Mol. Biol..

[191] Harm J., Fan Y., Brenner D. (2025). Navigating the metabolic landscape of regulatory T cells: From autoimmune diseases to tumor microenvironments. Curr. Opin. Immunol..

[192] Kempkes R.W.M., Joosten I., Koenen H.J.P.M., He X. (2019). Metabolic Pathways Involved in Regulatory T Cell Functionality. Front. Immunol..

[193] Miao Y., Zhang C., Yang L., Zeng X., Hu Y., Xue X., Dai Y., Wei Z. (2022). The activation of PPARgamma enhances Treg responses through up-regulating CD36/CPT1-mediated fatty acid oxidation and subsequent N-glycan branching of TbetaRII/IL-2Ralpha. Cell. Commun. Signal.

[194] Boss M., Kemmerer M., Brune B., Namgaladze D. (2015). FABP4 inhibition suppresses PPARgamma activity and VLDL-induced foam cell formation in IL-4-polarized human macrophages. Atherosclerosis.

[195] Cheng X., Li J., Guo D. (2018). SCAP/SREBPs are Central Players in Lipid Metabolism and Novel Metabolic Targets in Cancer Therapy. Curr. Top. Med. Chem..

[196] Sun G., Sun X., Li W., Liu K., Tian D., Dong Y., Sun X., Xu H., Zhang D. (2018). Critical role of OX40 in the expansion and survival of CD4 T-cell-derived double-negative T cells. Cell Death Dis..

[197] Pacella I., Procaccini C., Focaccetti C., Miacci S., Timperi E., Faicchia D., Severa M., Rizzo F., Coccia E.M., Bonacina F. (2018). Fatty acid metabolism complements glycolysis in the selective regulatory T cell expansion during tumor growth. Proc. Natl. Acad. Sci. USA.

[198] Raud B., Roy D.G., Divakaruni A.S., Tarasenko T.N., Franke R., Ma E.H., Samborska B., Hsieh W.Y., Wong A.H., Stuve P. (2018). Etomoxir Actions on Regulatory and Memory T Cells Are Independent of Cpt1a-Mediated Fatty Acid Oxidation. Cell Metab..

[199] Chapman N.M., Chi H. (2014). mTOR signaling, Tregs and immune modulation. Immunotherapy.

[200] Eid W., Dauner K., Courtney K.C., Gagnon A., Parks R.J., Sorisky A., Zha X. (2017). mTORC1 activates SREBP-2 by suppressing cholesterol trafficking to lysosomes in mammalian cells. Proc. Natl. Acad. Sci. USA.

[201] Yee P.P., Wei Y., Kim S., Lu T., Chih S.Y., Lawson C., Tang M., Liu Z., Anderson B., Thamburaj K. (2020). Neutrophil-induced ferroptosis promotes tumor necrosis in glioblastoma progression. Nat. Commun..

[202] Ugolini A., Tyurin V.A., Tyurina Y.Y., Tcyganov E.N., Donthireddy L., Kagan V.E., Gabrilovich D.I., Veglia F. (2020). Polymorphonuclear myeloid-derived suppressor cells limit antigen cross-presentation by dendritic cells in cancer. JCI Insight.

[203] Veglia F., Tyurin V.A., Blasi M., De Leo A., Kossenkov A.V., Donthireddy L., To T.K.J., Schug Z., Basu S., Wang F. (2019). Fatty acid transport protein 2 reprograms neutrophils in cancer. Nature.

[204] Liu R., Desai L.P. (2015). Reciprocal regulation of TGF-beta and reactive oxygen species: A perverse cycle for fibrosis. Redox Biol..

[205] Wang L., He G., Qi K., Yu L., Kong D., Gu J., Wang L. (2025). Lectin-like oxidized low-density lipoprotein receptor-1 reduces 5-FU sensitivity in gastric cancer cells via JAK/STAT/NOX4 axis. Biochem. Biophys. Res. Commun..

[206] Han B., Lin X., Hu H. (2024). Regulation of PI3K signaling in cancer metabolism and PI3K-targeting therapy. Transl. Breast Cancer Res..

[207] Vilbois S., Xu Y., Ho P. (2024). Metabolic interplay: Tumor macrophages and regulatory T cells. Trends Cancer.

[208] Deng H., Kan A., Lyu N., He M., Huang X., Qiao S., Li S., Lu W., Xie Q., Chen H. (2021). Tumor-derived lactate inhibit the efficacy of lenvatinib through regulating PD-L1 expression on neutrophil in hepatocellular carcinoma. J. Immunother. Cancer.

[209] Romero-Garcia S., Moreno-Altamirano M.M.B., Prado-Garcia H., Sanchez-Garcia F.J. (2016). Lactate Contribution to the Tumor Microenvironment: Mechanisms, Effects on Immune Cells and Therapeutic Relevance. Front. Immunol..

[210] DeBose-Boyd R.A., Ye J. (2018). SREBPs in Lipid Metabolism, Insulin Signaling, and Beyond. Trends Biochem. Sci..

[211] Su F., Huang S., Wei P., Hsu P., Li J., Su L., Hsieh Y., Hu C., Hsu J., Yang C. (2021). Redox sensor NPGPx restrains ZAP70 activity and modulates T cell homeostasis. Free Radic. Biol. Med..

[212] Li T., Han J., Jia L., Hu X., Chen L., Wang Y. (2019). PKM2 coordinates glycolysis with mitochondrial fusion and oxidative phosphorylation. Protein Cell.

[213] Bitorina A.V., Oligschlaeger Y., Shiri-Sverdlov R., Theys J. (2019). Low profile high value target: The role of OxLDL in cancer. Biochim. Biophys. Acta Mol. Cell Biol. Lipids.

[214] Pascual G., Avgustinova A., Mejetta S., Martin M., Castellanos A., Attolini C.S., Berenguer A., Prats N., Toll A., Hueto J.A. (2017). Targeting metastasis-initiating cells through the fatty acid receptor CD36. Nature.

[215] Schmitt B., Vicenzi M., Garrel C., Denis F.M. (2015). Effects of N-acetylcysteine, oral glutathione (GSH) and a novel sublingual form of GSH on oxidative stress markers: A comparative crossover study. Redox Biol..

[216] Traber M.G., Stevens J.F. (2011). Vitamins C and E: Beneficial effects from a mechanistic perspective. Free Radic. Biol. Med..

[217] Ma X., Bi E., Lu Y., Su P., Huang C., Liu L., Wang Q., Yang M., Kalady M.F., Qian J. (2019). Cholesterol Induces CD8(+) T Cell Exhaustion in the Tumor Microenvironment. Cell Metab..

[218] Schlaepfer I.R., Joshi M. (2020). CPT1A-mediated Fat Oxidation, Mechanisms, and Therapeutic Potential. Endocrinology.

[219] Chawla A., Boisvert W.A., Lee C.H., Laffitte B.A., Barak Y., Joseph S.B., Liao D., Nagy L., Edwards P.A., Curtiss L.K. (2001). A PPAR gamma-LXR-ABCA1 pathway in macrophages is involved in cholesterol efflux and atherogenesis. Mol. Cell.

[220] Shu Y., Qin M., Song Y., Tang Q., Huang Y., Shen P., Lu Y. (2020). M2 polarization of tumor-associated macrophages is dependent on integrin beta3 via peroxisome proliferator-activated receptor-gamma up-regulation in breast cancer. Immunology.

[221] Limburg P.J., Mahoney M.R., Ziegler K.L.A., Sontag S.J., Schoen R.E., Benya R., Lawson M.J., Weinberg D.S., Stoffel E., Chiorean M. (2011). Randomized phase II trial of sulindac, atorvastatin, and prebiotic dietary fiber for colorectal cancer chemoprevention. Cancer Prev. Res..

[222] Jo H., Kim S.T., Lee J., Park S.H., Park J.O., Park Y.S., Lim H.Y., Yu J.I., Park H.C., Choi D.H. (2023). A Phase II Study of Preoperative Chemoradiotherapy with Capecitabine Plus Simvastatin in Patients with Locally Advanced Rectal Cancer. Cancer Res. Treat..

[223] Lee J., Hong Y.S., Hong J.Y., Han S.W., Kim T.W., Kang H.J., Kim T.Y., Kim K., Kim S.H., Do I. (2014). Effect of simvastatin plus cetuximab/irinotecan for KRAS mutant colorectal cancer and predictive value of the RAS signature for treatment response to cetuximab. Invest New Drugs.

[224] Sharma R.A., Euden S.A., Platton S.L., Cooke D.N., Shafayat A., Hewitt H.R., Marczylo T.H., Morgan B., Hemingway D., Plummer S.M. (2004). Phase I clinical trial of oral curcumin: Biomarkers of systemic activity and compliance. Clin. Cancer Res..

[225] Wang F., He M., Xiao J., Zhang Y., Yuan X., Fang W., Zhang Y., Wang W., Hu X., Ma Z. (2022). A Randomized, Open-Label, Multicenter, Phase 3 Study of High-Dose Vitamin C Plus FOLFOX +/− Bevacizumab versus FOLFOX +/− Bevacizumab in Unresectable Untreated Metastatic Colorectal Cancer (VITALITY Study). Clin. Cancer Res..

[226] Hanson M.G.V., Ozenci V., Carlsten M.C.V., Glimelius B.L., Frodin J.A., Masucci G., Malmberg K., Kiessling R.V.R. (2007). A short-term dietary supplementation with high doses of vitamin E increases NK cell cytolytic activity in advanced colorectal cancer patients. Cancer Immunol. Immunother..

[227] Komatsu Y., Yoshino T., Yamazaki K., Yuki S., Machida N., Sasaki T., Hyodo I., Yachi Y., Onuma H., Ohtsu A. (2014). Phase 1 study of efatutazone, a novel oral peroxisome proliferator-activated receptor gamma agonist, in combination with FOLFIRI as second-line therapy in patients with metastatic colorectal cancer. Invest. New Drugs.

[228] Diakowska D., Grabowski K., Nienartowicz M., Zarebski P., Fudalej K., Markocka-Maczka K. (2015). Circulating Oxidized Low-Density Lipoproteins and Antibodies against Oxidized Low-Density Lipoproteins as Potential Biomarkers of Colorectal Cancer. Gastroenterol. Res. Pract..

